# The CsWRKY50-*CsREM1*-*CsTSⅠ* module inhibits theanine biosynthesis in tea plants under drought stress

**DOI:** 10.1093/plphys/kiaf437

**Published:** 2025-09-25

**Authors:** Shenyuan Ye, Linlin Li, Ping Li, Xinzhuan Yao, Qi Zhao, Shiyu Tian, Tong Li, Yihe Jiang, Zhenkedai Yuan, Yu Chen, Qi-hong Zou, Shi-yu Zhang, Yue Wan, Chao Xu, Hui Hu, Zifan Yang, Chao Luo, Li-Tang Lu

**Affiliations:** College of Tea Science and the Key Laboratory of Plant Resources Conservation and Germplasm Innovation in Mountainous Region (Ministry of Education), Guizhou University, Guiyang 550025, China; College of Tea Science and the Key Laboratory of Plant Resources Conservation and Germplasm Innovation in Mountainous Region (Ministry of Education), Guizhou University, Guiyang 550025, China; College of Tea Science and the Key Laboratory of Plant Resources Conservation and Germplasm Innovation in Mountainous Region (Ministry of Education), Guizhou University, Guiyang 550025, China; College of Life Science, Guizhou University, Guiyang 550025, China; College of Tea Science and the Key Laboratory of Plant Resources Conservation and Germplasm Innovation in Mountainous Region (Ministry of Education), Guizhou University, Guiyang 550025, China; College of Life Science, Guizhou University, Guiyang 550025, China; College of Tea Science and the Key Laboratory of Plant Resources Conservation and Germplasm Innovation in Mountainous Region (Ministry of Education), Guizhou University, Guiyang 550025, China; College of Life Science, Guizhou University, Guiyang 550025, China; College of Life Science, Guizhou University, Guiyang 550025, China; College of Tea Science and the Key Laboratory of Plant Resources Conservation and Germplasm Innovation in Mountainous Region (Ministry of Education), Guizhou University, Guiyang 550025, China; College of Tea Science and the Key Laboratory of Plant Resources Conservation and Germplasm Innovation in Mountainous Region (Ministry of Education), Guizhou University, Guiyang 550025, China; College of Life Science, Guizhou University, Guiyang 550025, China; College of Life Science, Guizhou University, Guiyang 550025, China; College of Tea Science and the Key Laboratory of Plant Resources Conservation and Germplasm Innovation in Mountainous Region (Ministry of Education), Guizhou University, Guiyang 550025, China; College of Life Science, Guizhou University, Guiyang 550025, China; Huaneng Clean Energy Research Institute, Beijing 102209, China; Huaneng Guizhou Clean Energy Branch, Guiyang 550081, China; Huaneng Guizhou Clean Energy Branch, Guiyang 550081, China; Huaneng Clean Energy Research Institute, Beijing 102209, China; College of Tea Science and the Key Laboratory of Plant Resources Conservation and Germplasm Innovation in Mountainous Region (Ministry of Education), Guizhou University, Guiyang 550025, China; College of Forestry, Guizhou University, Guiyang 550025, China; College of Tea Science and the Key Laboratory of Plant Resources Conservation and Germplasm Innovation in Mountainous Region (Ministry of Education), Guizhou University, Guiyang 550025, China; College of Life Science, Guizhou University, Guiyang 550025, China

## Abstract

Drought affects theanine biosynthesis in tea (*Camellia sinensis* L.) plants, but how drought stress affects the associated regulatory mechanisms remains unclear. Here, we explored the molecular regulatory network governing theanine biosynthesis under drought stress. Prolonged drought stress significantly reduced theanine content and the expression of *C. sinensis* theanine synthase *Ⅰ* (*CsTSⅠ*) in tea plant leaves. We employed yeast 1-hybrid (Y1H) screening using the *CsTSⅠ* promoter as bait to identify transcription factors regulating *CsTSⅠ* transcription. Analysis of the drought stress transcriptome facilitated identification of the transcription factor *C. sinensis* REPRODUCTIVE MERISTEM 1 (CsREM1), whose encoding gene had Fragments Per Kilobase of exon model per Million mapped fragments values significantly correlated with theanine content and *CsTSⅠ* expression. Further experiments confirmed that CsREM1 can directly bind to the promoter region of *CsTSⅠ*, thereby positively regulating its transcription and enhancing theanine biosynthesis. To investigate the molecular mechanisms by which drought conditions inhibit theanine biosynthesis, we employed Weighted Gene Co-expression Network Analysis (WGCNA) and identified the transcription factor CsWRKY50. Our findings indicate that as the duration of drought stress increases, *CsWRKY50* expression is upregulated. CsWRKY50 directly interacts with the promoter region of *CsREM1*, negatively regulating its transcription and, consequently, its effect on *CsTSI*. Ultimately, downregulation of *CsTSⅠ* transcription leads to diminished theanine biosynthesis. Overall, our findings indicate that the CsWRKY50-*CsREM1*-*CsTSⅠ* module is crucial for regulating theanine biosynthesis under drought stress.

## Teaser

CsWRKY50 and CsREM1 play key roles in inhibiting theanine biosynthesis under drought stress.

## Introduction

Theanine, catechins, and caffeine are 3 highly important secondary metabolites found in tea plants [*Camellia sinensis* (L.) O. Ktze]. Theanine is the main contributor to the umami flavor present in tea and is also one of the important indicators of the quality of different tea plant varieties (Yamaguchi and Ninomiya 2000; Mata-Bilbao et al. 2007; Tai et al. 2018). Theanine has been studied since the 1950s and was first identified in water extracts of tea leaves (Sakato 1950). More recently, *C. sinensis* theanine synthase Ⅰ (CsTSⅠ) has been identified as a key enzyme involved in theanine synthesis. The specific enzymatic reactions facilitated by CsTSⅠ involve the conversion of glutamic acid (Glu) and ethylamine (EA) into theanine (Sasaoka et al. 1963, 1965). In tea plants, ethylamine is produced from alanine via a decarboxylation reaction catalyzed by alanine decarboxylase. Furthermore, tracer experiments using [U-^14^C]-alanine and [1-^14^C]-ethylamine have confirmed that Glu and EA can act as substrates for the theanine synthesis catalyzed by CsTSⅠ (Deng et al. 2009). More recent research has demonstrated that theanine can be successfully synthesized in vitro using the recombinant protein CsTSⅠ produced by *Escherichia coli* (*E. coli*), along with Glu and EA as substrates. Moreover, overexpression of *CsTSⅠ* in *Arabidopsis thaliana*, combined with the application of exogenous EA, has enabled the successful synthesis of theanine in a nontea plant (She et al. 2022). In addition to CsTSⅠ, several other key enzymes are involved in the theanine synthesis pathway, including CsAlaDC, CsGS, and CsGGT, among others (Zhu et al. 2021; She et al. 2022; Chang et al. 2024).

Transcription factors (TFs) are major regulators of gene expression and play a crucial role in the molecular regulation of theanine synthesis (Liu et al. 2024; Wang et al. 2025). For example, CsMYB73 has been found to interact with the promoters of *CsGGT2* and *CsGGT4*, where it exerts a negative regulatory effect on theanine synthesis, causing reduced accumulation in the new shoots of *C. sinensis* (Chang et al. 2024). Conversely, CsTCP2 upregulates theanine synthesis via interacting with the *CsGS* promoter (Mao et al. 2025). In addition, CsGTAT17 promotes theanine synthesis by interacting with the promoter region of *CsAlaDC* (Gao et al. 2025). The synthesis of theanine is further influenced by interplay of TF modules that are known to be associated with abiotic stress. For instance, gibberellin enhances theanine synthesis by downregulating the expression of CsWRKY71, which alleviates specific repression of *CsTSⅠ* that is mediated by CsWRKY71 (Xiang et al. 2025). Furthermore, CsWRKY53 and CsWRKY40 work synergistically via the abscisic acid signaling pathway to regulate theanine hydrolysis during the withering process of tea production (Haiyan et al. 2024). Overall, the CsSPX3-*CsPHL7*-*CsTSⅠ*/*CsGS1* module elucidates the molecular mechanism underlying the phosphorus inhibition of theanine synthesis (Chen et al. 2024a, 2024b), while the MYB TF family member CsMOF1 in tea plants responds to drought stress and can directly bind to the promoter region of *CsGS1* to inhibit its expression, thereby negatively regulating theanine synthesis (Chen et al. 2024a, 2024b).

Beyond the regulatory role TFs play regarding the synthesis of theanine, environmental factors also exert an important regulatory influence. For example, high temperature stress (Li et al. 2016, 2018; Liu et al. 2017), cold stress (Wang et al. 2022), and saline-alkali stress are all known to significantly reduce theanine levels (Wan et al. 2024). Conversely, shading treatments can enhance tea plant accumulation and synthesis of theanine (Dai et al. 2015; Ji et al. 2018; Hu et al. 2024). More recent research has indicated that global warming is leading to increasingly severe drought (Ault 2020).

Drought stress has also been found to profoundly affect the growth of tea plants. It causes cell membrane damage and compromises normal cellular functions; affects the ABA signaling pathway, leading to excessive ABA accumulation; and also reduces the levels of secondary metabolites such as total polyphenols, free amino acids, and caffeine, along with downregulation of genes involved in secondary metabolite synthesis (Ault 2020; Chaeikar et al. 2020; Christian et al. 2021; Zhang et al. 2022; Yue et al. 2023). Moreover, recent research has indicated that extended periods of drought stress can result in reduced theanine levels and downregulation of theanine synthesis-related gene expression while simultaneously upregulating the expression of TF family members such as WRKY, bHLH, and MYB (Wang et al. 2016a, 2016b; Zhang et al. 2017; Chen et al. 2024a, 2024b). Nevertheless, to date, there is limited information regarding the molecular mechanisms responsible for inhibiting the synthesis of theanine in tea plants under drought stress.

In this study, we report that CsWRKY50 and CsREM1 are crucial TFs that govern the molecular regulatory mechanisms by which drought stress inhibits theanine synthesis. Under drought stress conditions, *CsWRKY50* mRNA is upregulated. The resulting CsWRKY50 protein interacts with the promoter region of *CsREM1*, and high levels of *CsWRKY50* expression cause downregulation of *CsREM1* transcription. The downregulation of *CsREM1* expression in turn reduces its positive regulatory influence on *CsTSⅠ*, which leads to lower expression levels of *CsTSⅠ* and the subsequent inhibition of theanine synthesis. Thus, according to our results, the CsWRKY50-*CsREM1*-*CsTSⅠ* module plays a key role in the regulation of the molecular mechanism by which drought inhibits theanine synthesis.

## Results

### Drought stress reduces theanine content in tea plant leaves and downregulates genes involved in theanine synthesis

**Figure 1.**
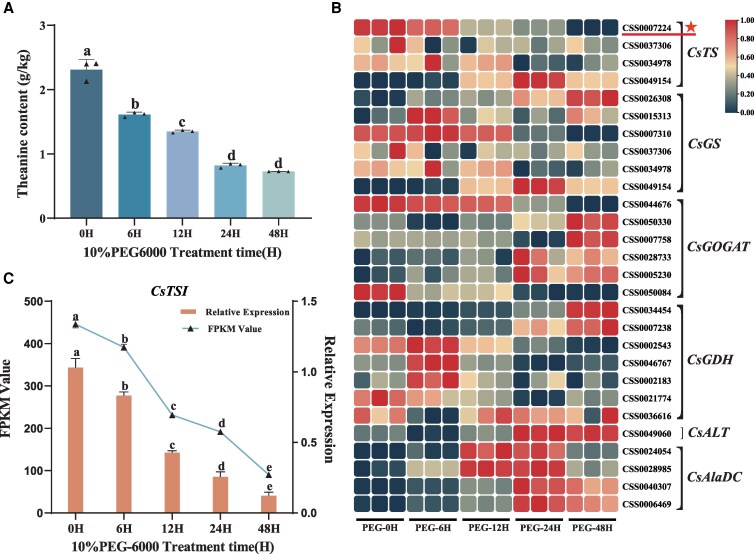
Changes in theanine content in tea plant leaves and an analysis of genes related to theanine synthesis in tea plants under drought stress. **A)** Changes in theanine content of tea plant leaves under drought stress. **B)** RNA-seq transcriptomic map of theanine synthesis pathway-related genes of tea plants under drought stress. **C)** Relative expression level of *CsTSⅠ* and FPKM values of leaves of tea plants under drought stress. Error bars represent the mean ± SE of 3 biological replicates. SE, standard error. Values in **A)** and **C)** indicate the mean ± SE of 3 biological replicates (*n* = 3), and the statistical significance of differences in mean was determined using ANOVA and Tukey's HDS tests. Different lowercase letters indicate the presence of statistically significant differences between samples (*P* < 0.05).

Firstly, we added polyethylene glycol 6000 (PEG-6000) to the hydroponic nutrient solution administered to tea plants under cultivation (Supplementary Table S1) to simulate drought stress. This simulated drought treatment resulted in a diminished concentration of theanine in tea plant leaves as the duration of the drought stress increased (Fig. 1A). Next, we conducted transcriptomic sequencing of tea plant leaves subjected to drought stress and analyzed the Fragments Per Kilobase of exon model per Million mapped fragments (FPKM) values of genes known to be involved in theanine synthesis pathways. This analysis revealed a strong and positive correlation between the expression of theanine synthase *CsTSⅠ* (CSS007224) and theanine content, especially when compared with other *CsTSs* (Fig. 1B and Supplementary Table S2). Next, we confirmed this finding by analyzing reverse transcription quantitative polymerase chain reaction (RT-qPCR) results for *CsTSⅠ* (Fig. 1C), by performing a Pearson's correlation analysis of the FPKM values of theanine synthesis-related genes, and by quantifying the content of theanine in plants under drought stress (Supplementary Fig. S1). Therefore, we speculated that *CsTSⅠ* plays a key role in theanine synthesis in tea plant leaves under drought stress.

### Discovery of a candidate TF that may bind *CsTSⅠ*

Next, we examined the TFs that may regulate theanine synthesis. To do so, we first generated a *ProCsTSⅠ*-pAbAi vector and transformed it into yeast 1-hybrid (Y1H) Gold chemically competent cells for self-activation verification experiments. The empty pGADT7 vector was introduced into *ProCsTSⅠ*-pAbAi competent cells, followed by plating on SD/-Leu and SD/-Leu/Aureobasidin A(AbA). No colonies were detected at SD/-Leu/600 ng/mL AbA, confirming that this concentration represents the minimum inhibitory level for AbA (Supplementary Fig. S2A).

Next, the library plasmid was transformed into *ProCsTSⅠ*-pAbAi competent cells, which were then spread onto SD/-Leu/600 ng/mL AbA plates for 3 to 5 d of cultivation. Positive clones from the initial screening were then transferred to fresh SD/-Leu/600 ng/mL AbA plates for further verification (Supplementary Fig. S2B) via PCR and agarose gel electrophoresis analyses. Overall, our results reported that as many as 15 TFs were capable of binding to the *CsTSⅠ* promoter (Supplementary Fig. S2C).

### Screening of positive clones and identification of the key TF-*CsREM1*

**Figure 2.**
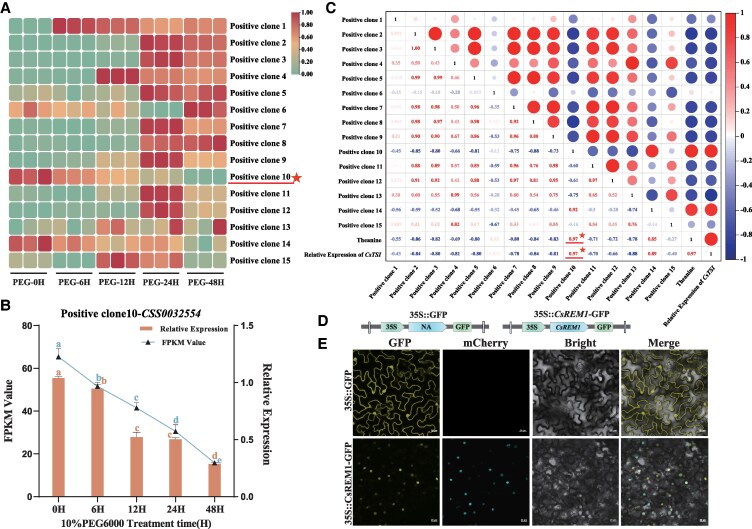
Screening and subcellular localization of *CsREM1*. **A)** An RNA-seq transcriptome map of 15 positive clones screened by Y1H experiment. Transcriptomic data reflects tea plant leaves subjected to drought stress. **B)** Relative expression and FPKM value of Positive Clone 10 (*CsREM1)* in tea plants under drought stress. **C)** Pearson's correlation analysis of the 15 positive clones regarding theanine content and *CsTSⅠ* expression in tea plants under drought stress. **D)** Vector for subcellular localization of *CsREM1*. **E)** Subcellular localization of CsREM1-GFP in leaves of *N. benthamiana*. Scale bar = 20 *μ*m. Error bars represent the mean ± SE of 3 biological replicates. SE, standard error of the mean. Values shown in **B)** reflect the mean ± SE of 3 biological replicates (*n* = 3). Statistical significance of mean differences was determined by ANOVA and Tukey's HSD tests. Different lowercase letters indicate that differences among sample means are statistically significant (*P* < 0.05).

Next, we conducted genetic analysis of the 15 positive clones reported above by BLAST querying the National Center for Biotechnology Information (NCBI) and Tea Plant Information Archive (TPIA) databases to obtain corresponding gene IDs. Subsequently, we analyzed the FPKM values of all clones in the drought transcriptome and identified a TF, Positive Clone 10, that exhibited a trend similar to that of theanine content and the relative expression of *CsTSⅠ* in tea plant leaves subjected to drought stress (Fig. 2A). Further RT-qPCR results also confirmed this identification (Fig. 2B).

We then conducted Pearson's correlation analyses on the relationship between theanine content, *CsTSⅠ* expression in tea plants subjected to drought stress, and the FPKM values of 15 positive clones. These results indicated that Positive Clone 10 demonstrated strong and significant correlations with theanine content (*R* = 0.97) and *CsTSⅠ* expression (*R* = 0.97) (Fig. 2C). We therefore speculated that may be TF linked to transcriptional regulation of *CsTSⅠ*. Based on conserved domain analysis and inquiries from the NCBI and TPIA databases, we located XP_028101879 (NCBI database accession number) or CSS0032554 (gene ID in the TPIA database for the ShuchaZao2 genome) on the seventh chromosome of *C. sinensis* (Supplementary Fig. S3B) and found that this gene belongs to the B3 family (Supplementary Fig. S3A). Next, we performed a phylogenetic analysis to comparing this TF to clearly annotated members of the B3 gene superfamily found in *A. thaliana* and rice (*Oryza sativa*). These results indicated that CSS0032554 clusters in the same branch as *A. thaliana* REPRODUCTIVE MERISTEM 1 (AtREM1), so we named this TF *C. sinensis* REPRODUCTIVE MERISTEM 1(CsREM1) (Supplementary Fig. S3C) and refer to it by this name hereafter.

To elucidate the subcellular localization of *CsREM1*, we generated a fusion protein expression vector that combined *CsREM1* with a GFP (35S::*CsREM1*-GFP) (Fig. 2D). We then transiently transformed 35S::*CsREM1*-GFP, 35S::*H2B*-mCherry, and an empty vector into the leaves of *Nicotiana benthamiana* plants. We found that the CsREM1-GFP recombinant plasmid induced fluorescence exclusively in the nucleus, whereas the empty vector showed fluorescence signals in both cytoplasm and nucleus. Taken together, these data indicate that *CsREM1* is localized to the nucleus (Fig. 2E).

### CsREM1 binds to the promoter of *CsTSⅠ* and promotes *CsTSⅠ* transcription

**Figure 3.**
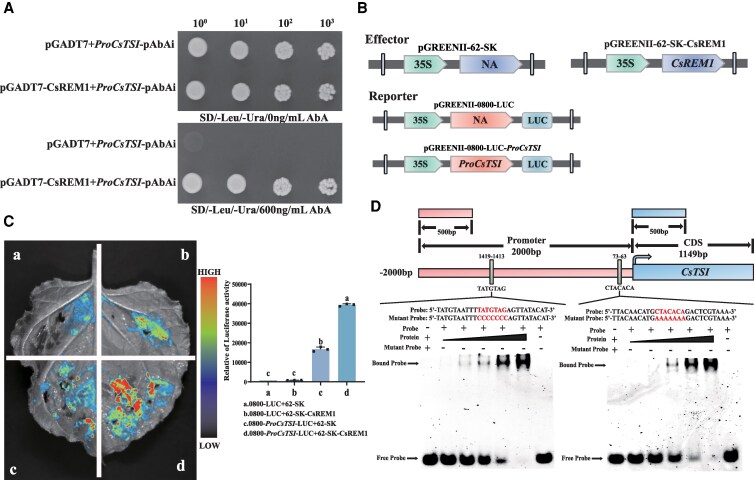
CsREM1 binds to the promoter of *CsTSⅠ* and positively regulates its expression. **A)** Y1H assays reveal an interaction between CsREM1 and the *CsTSⅠ* promoter region. **B)** Structure of the reporter and effector used for the dual luciferase assay. **C)** A dual luciferase expression assay shows that CsREM1 enhances the expression of *CsTSⅠ*. **D)** EMSA results show that the CsREM1-GST fusion protein binds to TATGTAG and CTACACA DNA sequence motifs within the *CsTSⅠ* promoter region. Error bars represent mean ± SE of 3 biological replicates. SE, standard error of the mean. Values shown in **C)** represent the mean ± SE of 3 biological replicates (*n* = 3). The statistical significance of differences in group means was determined by ANOVA and Tukey's HSD tests. Different lowercase letters indicate statistically significant differences between sample means (*P* < 0.05). SD, synthetic dextrose minimal medium; ABA, Aureobasidin A; SD/-Leu/-Ura, SD medium lacking leucine and uracil; SD/-Leu/-Ura/AbA, SD medium containing ABA but lacking leucine and uracil, with the number before ABA representing the concentration of ABA.

To clarify how CsREM1 regulates *CsTSⅠ*, we first performed a Y1H experiment. To do so, we initially cultured yeast cells containing pGADT7 and *ProCsTSⅠ*-pAbAi on a SD/-Leu screening medium to determine the optimal concentration of AbA required for inhibition of self-activation of the bait (Supplementary Fig. S4A). The Y1H experimental results indicated that yeast harboring pGADT7 and *ProCsTSⅠ*-pAbAi exhibited normal growth on SD/-Leu/-Ura medium at an AbA concentration of 0 ng/mL, but failed to grow on plates containing SD/-Leu/-Ura/600 ng/mL ABA. In contrast, yeast containing pGADT7-*CsREM1* and *ProCsTSⅠ*-pAbAi demonstrated robust growth on both SD/-Leu/-Ura and SD/-Leu/-Ura/600 ng/mL ABA, therefore suggesting that CsREM1 can interact with the promoter region of *CsTSⅠ* in vivo (Fig. 3A).

To further validate that CsREM1 participates in the transcriptional regulation of *CsTSⅠ*, we performed a dual luciferase expression assay. To do so, the coding DNA sequence (CDS) of *CsREM1* was cloned into a pGREENII 62-SK vector, while the promoter sequence of *CsTSⅠ* was inserted into a pGREENII0800-LUC vector (Fig. 3B). We then conducted another transient expression assay in tobacco leaves. These results demonstrated that the luminescence intensity of 35S::*CsREM1* coexpressed with *ProCsTSⅠ*::LUC in tobacco leaves was 2.5 times greater than that observed for *ProCsTSⅠ*::LUC alone (Fig. 3C).

Next, to locate the binding site of CsREM1 within the *CsTSⅠ* promoter region, we performed an electrophoretic mobility shift assay (EMSA). Briefly, a CsREM1-GST fusion protein (Supplementary Fig. S5, A and B) was incubated with *CsTSⅠ* promoter sequences containing various DNA fragments (i.e. probes), which were then analyzed via EMSA. These results demonstrated that CsREM1 specifically bound to TATGTAG and CTACACA DNA sequence motifs with in the *CsTSⅠ* promoter region (Fig. 3D).

Overall, these findings indicate that CsREM1 can directly bind to the promoter region of *CsTSⅠ* and enhances the transcriptional of *CsTSⅠ* activity.

### CsREM1 upregulates *CsTSⅠ* transcription and promotes theanine synthesis

Next, we used virus-induced gene signaling (VIGS) to downregulate *CsREM1* in “Fuding dabaicha” to elucidate whether *CsREM1* influences theanine synthesis. After 40 d, we harvested the leaves of silenced *CsREM1* (pTRV2-*CsREM1*), wild type (WT), and pTRV1 + pTRV2 (pTRV2) tea plants.

**Figure 4.**
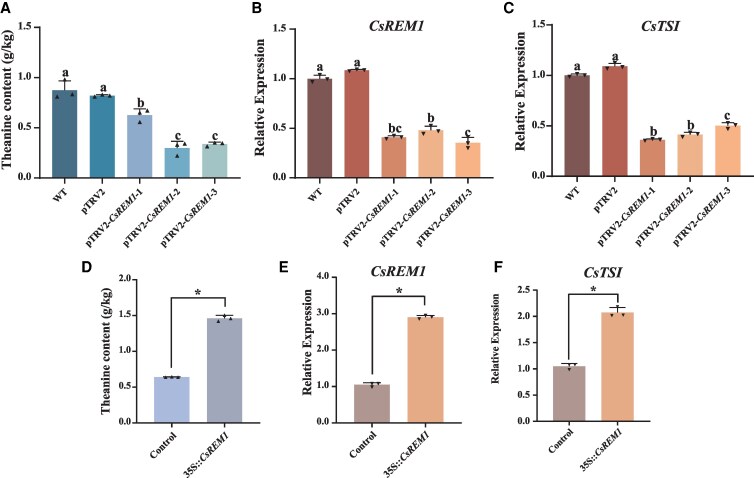
*CsREM1* regulates theanine synthesis in tea plants. **A)** Changes in theanine content in WT, pTRV2, and pTRV2-*CsREM1* lines after silencing of *CsREM1* in tea plant leaves by VIGS. Also shown are the relative expression levels of **B)**  *CsREM1* and **C)**  *CsTSⅠ* in *CsREM1*-silenced plants. **D)** Transient overexpression of *CsREM1* in tea plant leaves increases theanine content. The relative expression levels of **E)**  *CsREM1* and **F)**  *CsTSⅠ* in plants overexpressing *CsREM1*. Error bars represent the mean ± SE of the 3 biological replicates. SE, standard error of the mean; TRV, tobacco rattle virus; VIGS, virus-induced gene signaling. Values in **A** to **C)** are the mean ± SE of 3 biological replicates (*n* = 3), and statistical significance was determined by ANOVA and Tukey's HSD tests. Different lowercase letters indicate statistically significant differences between sample means (*P* < 0.05). Values in **D** to **F)** represent the mean ± SE of 3 biological replicates (*n* = 3), and the statistical significance of differences in group means was determined by Student's *t* test (*P < 0.05).

RT-qPCR analyses demonstrated effective silencing of *CsREM1* in VIGS-treated tea plants, pTRV2-*CsREM1*-1, pTRV2-*CsREM1*-2, and pTRV2-*CsREM1*-3, in which the relative expression levels of *CsREM1* were significantly reduced by 60%, 50%, and 65%, respectively (Fig. 4B). Furthermore, we also observed a significant reduction in the expression of *CsTSⅠ* in *CsREM1*-silenced lines (Fig. 4C). Furthermore, HPLC analysis showed that the theanine content in pTRV2-*CsREM1* was significantly lower than the WT and pTRV2 lines, with pTRV2-*CsREM1*-2, and pTRV2-*CsREM1*-3 showing the most significant decreases in theanine content, with reductions of 60% and 55%, respectively (Fig. 4A).

Nest, we performed *Agrobacterium*-mediated transient overexpression by injecting *Agrobacterium tumefaciens* harboring pCAMBIA2301-35S-*CsREM1* into the leaves of tea plants. After 4 d, plant samples were collected for subsequent transcriptomic analysis. Here, RT-qPCR results confirmed substantial upregulation of *CsREM1* expression in overexpression tea plant leaves relative to a control (Fig. 4E). Notably, we also observed significant upregulation of *CsTSⅠ* expression in overexpression lines (Fig. 4F). Moreover, HPLC analysis confirmed that the levels of theanine in leaves overexpressing *CsREM1* were elevated by 200% relative to control samples (Fig. 4D). Taken together, our results show that in tea plants, CsREM1 expression caused upregulation of *CsTSⅠ* transcription, thereby promoting theanine synthesis in tea plant leaves.

### Weighted Gene Co-expression Network Analysis transcriptomic analysis of the leaves of tea plants under drought stress

So far, we have established that *CsREM1* positively regulates theanine synthesis in tea plant leaves. However, under drought stress, theanine content declined, and the expression levels of both *CsREM1* and *CsTSⅠ* decreased. We hypothesize that under drought stress, TF-based regulatory patterns may hinder the transcription of *CsREM1*, thereby influencing the transcription of *CsTSⅠ* and subsequently affecting theanine synthesis. Thus, we subsequently conducted a Weighted Gene Co-expression Network Analysis (WGCNA) analysis on the drought stress transcriptome to determine whether this hypothesis was correct.

**Figure 5.**
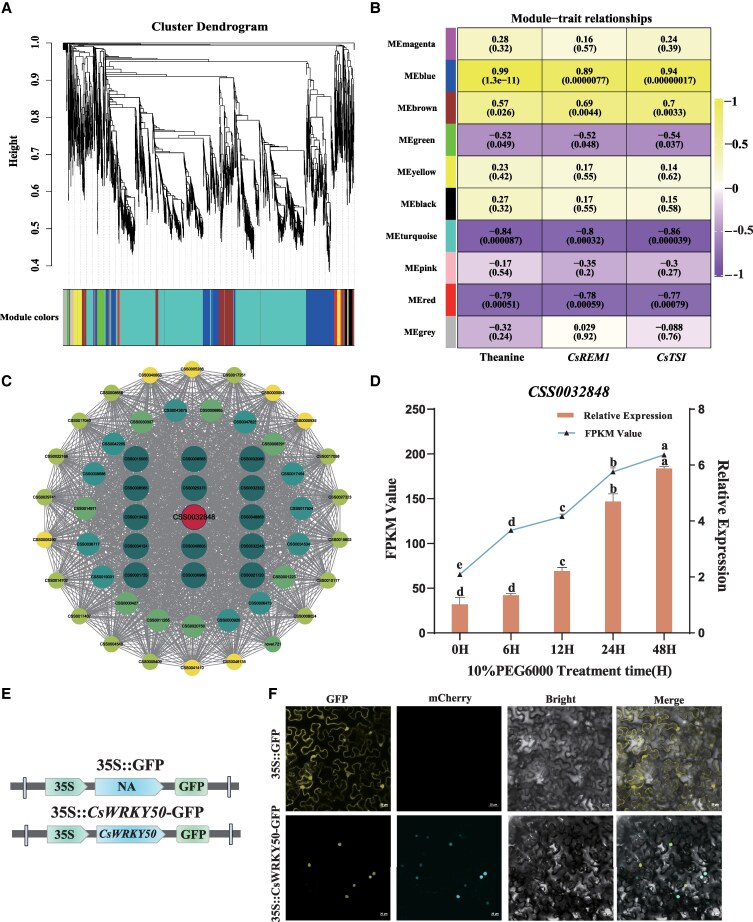
The origin, definition, and subcellular localization of *CsWRKY50*. **A)** Clustering tree diagram of genes of interest. **B)** Correlation heat map between modules and traits. **C)** Gene coexpression networks of genes from the MEblue module. **D)** Relative expression and FPKM value of *CsWRKY50* in tea plants subjected to drought stress. **E)** Vector for the subcellular localization of *CsWRKY50*. **F)** Subcellular localization of CsWRKY50-GFP in the leaves of *N. benthamiana*. Scale bar = 20 *μ*m. Error bars represent the mean ± SE of the 3 biological replicates. SE, standard error of the mean. Values in **D)** represent the mean ± SE of 3 biological replicates (*n* = 3), and statistical significance was determined by ANOVA and Tukey's HSD tests. Different lowercase letters indicate statistically significant differences between sample means (*P* < 0.05).

Our WGCNA analysis categorized genes into 10 coexpression modules, designated in Fig. 5 by distinct colors, including magenta, blue, brown, green, yellow, black, turquoise, pink, red, and gray (Fig. 5A). Next, we analyzed the degree of correlation between theanine content, *CsREM1* expression, *CsTSⅠ* expression, and module epigenetic genes in plants subjected drought stress. As shown in Fig. 5B, we found that the blue module was significantly correlated (*R* > 0.8; *P* < 0.01) with changes in theanine content, *CsREM1* relative expression, and the relative expression of *CsTSⅠ.* Accordingly, we then constructed a coexpression network based on the weighted values of genes present in the blue module. Hub genes were first selected according to their degree of connectivity within the network. In the blue module, we identified 15 core genes, among which was the core TF CSS0032848 (Fig. 5C). To determine whether CSS0032848 was indeed by plant exposure to drought conditions, we analyzed its FPKM value in the drought stress transcriptome and performed RT-qPCR analysis of drought-treated plant samples. These results confirmed that its expression was significantly upregulated as the drought duration was prolonged (Fig. 5D).

According to the conserved domain analysis of sequences in the NCBI and TPIA databases, CSS0032848 belongs to the WRKY gene family (Supplementary Fig. S3D) and is located on Chromosome 14 of the *C. sinensis* genome (Supplementary Fig. S3E). Next, we performed phylogenetic tree analysis using data from the WRKY gene family of *Arabidopsis* and found that CSS0032848 belonged to the same branch as AtWRKY50. We therefore named CSS0032848 as CsWRKY50 (Supplementary Fig. S3F) and refer to it hereafter by that name. Next, to elucidate the subcellular localization of CsWRKY50, we generated the fusion protein expression vector −35S::*CsWRKY50*-GFP (Fig. 5E). Subsequently, we transformed both this fusion construct and an empty vector containing only GFP into the leaves of tobacco plants. We found that the CsWRKY50-GFP recombinant plasmid-induced fluorescence exclusively in the nucleus, whereas the empty vector showed fluorescent signals in both the cytoplasm and nucleus. These results, therefore, indicate that *CsWRKY50* is localized within the nucleus (Fig. 5F).

### CsWRKY50 downregulates the transcription of *CsREM1* and *CsTSⅠ* and inhibits theanine synthesis

Next, we considered whether CsWRKY50 screened by WGCNA analysis as described above could regulate the transcription of *CsREM1*. This would in turn affect the transcriptional regulation of *CsTSⅠ* by *CsREM1*, potentially leading to the inhibition of theanine synthesis. To validate this hypothesis, we used VIGS to downregulate the expression of *CsWRKY50* in “Fuding dabaicha” tea plants. After 40 d, we harvested leaves from silenced *CsWRKY50* (pTRV2-*CsWRKY50*), WT, and pTRV2 tea plants.

**Figure 6.**
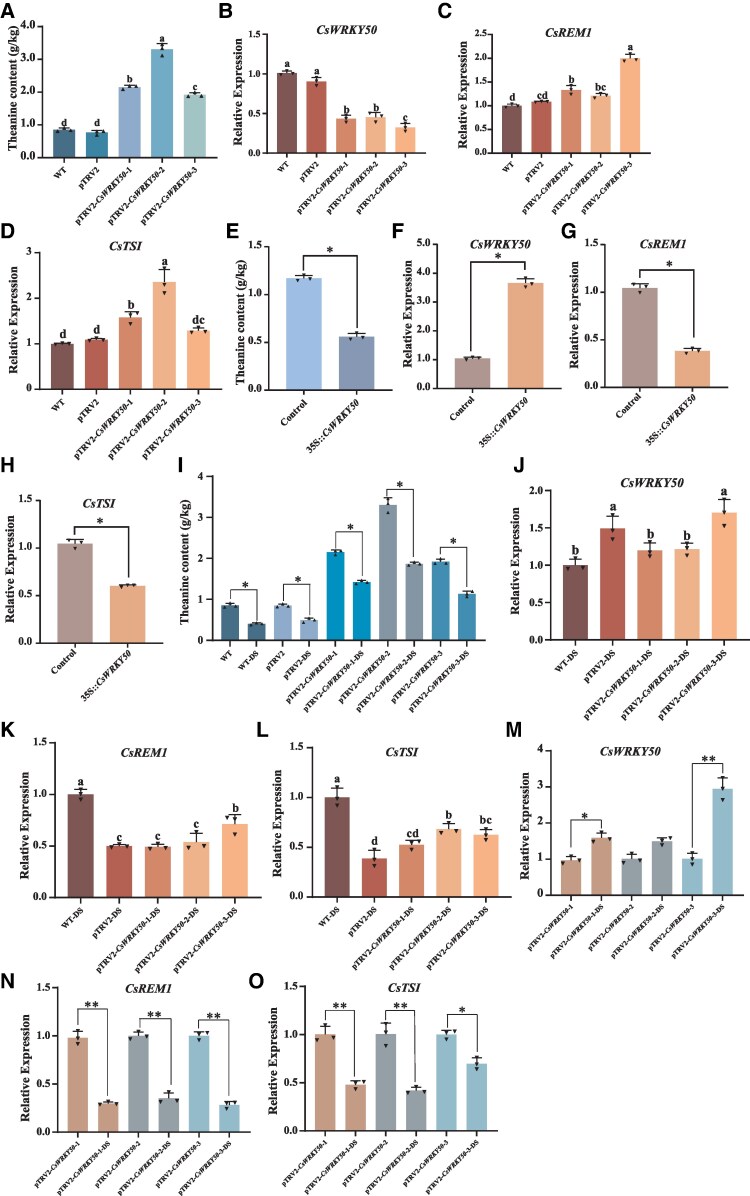
The regulation of CsWRKY50 on *CsREM1*, *CsTSⅠ*, and theanine synthesis. **A)** Changes in theanine content of tea plant leaves in WT, pTRV2, and pTRV2-*CsWRKY50* after silencing *CsWRKY50* by VIGS. Also shown are the relative expression levels of **B)**  *CsWRKY50*, **C)**  *CsREM1*, and **D)**  *CsTSⅠ* in *CsWRKY50*-silenced plants. **E)**. Transient overexpression of *CsWRKY50* in tea plant leaves increased theanine content. The relative expression levels of **F)**  *CsWRKY50*, **G)**  *CsREM1*, and **H)**  *CsTSⅠ* in *CsWRKY50-*overexpressing tea plants. **I)** Changes in theanine content in WT, pTRV2, and pTRV2-*CsWRKY50* in tea plants subjected to drought stress. The relative expression levels of **J)**  *CsWRKY50*, **K)**  *CsREM1*, and **L)**  *CsTSⅠ* in *CsWRKY50*-silenced tea plants subjected to drought stress. Also shown are comparisons of the expression levels of **M)**  *CsWRKY50*, **N)**  *CsREM1*, and **O)**  *CsTSⅠ* in *CsWRKY50*-silenced lines under drought stress and *CsWRKY50-*silenced lines that were not subjected to drought stress. Error bars represent mean ± SE of 3 biological replicates. SE, standard error of the mean. TRV, tobacco rattle virus; VIGS, virus-induced gene signaling; DS, drought stress. Values in **A** to **D)** and **J** to **L)** represent the mean ± SE of 3 biological replicates (*n* = 3). Statistical significance of differences in group means was determined by ANOVA and Tukey's HSD tests. Different lowercase letters indicate that there are statistically significant differences between the sample group means (*P* < 0.05). Values in **E** to **G)**, **H** and **I)**, and **M** to **O)** represent the mean ± SE of 3 biological replicates (*n* = 3), and the statistical significance of differences in group means was determined by Student's *t* test (*P < 0.05; **P < 0.01).

RT-qPCR analyses of these leaf samples demonstrated that *CsWRKY50* was effectively silenced in VIGS-treated tea plants (Fig. 6B). Furthermore, the expressions of *CsTSⅠ* and *CsREM1* were both strongly elevated in pTRV2-*CsWRKY50* samples (Fig. 6, C and D). Furthermore, in pTRV2-*CsWRKY50*-1, -2, and -3, the relative expression of *CsWRKY50* was significantly reduced by 57%, 50%, and 68%, respectively. A subsequent HPLC analysis indicated that relative to lines that were not given VIGS treatment, the theanine content of pTRV2-*CsWRKY50* was significantly increased, with pTRV2-*CsWRKY50*-1, -2, and -3 showing significant increases of 130%, 270%, and 120%, respectively (Fig. 6A).

Next, we performed *Agrobacterium*-mediated transient overexpression experiments by injecting pCAMBIA2301-35S-*CsWRKY50* into tea plant leaves. For these experiments, we used pCAMBIA2301-35S as a control. As illustrated in Fig. 6F, the expression levels of *CsWRKY50* were strongly elevated in OE-*CsWRKY50* lines. Moreover, this increase was associated with a substantial reduction of 58% in theanine content (Fig. 6E), as well as with significant downregulation of *CsREM1* and *CsTSⅠ* expression (Fig. 6, G and H).

To further confirm that *CsWRKY50* regulates *CsREM1*, *CsTSⅠ*, and theanine, we subjected VIGS-treated plants to drought stress for 24 h in a culture medium containing 10% PEG-6000. These results showed that the theanine content of WT-DS (drought stress), pTRV2-DS, and pTRV2-*CsWRKY50*-1,2,3-DS plants all decreased significantly following 24 h drought stress relative to lines that were not subjected to drought stress treatments. Moreover, the theanine content in WT plants decreased by 55%, in pTRV2 by 47%, and in pTRV2-CsWRKY50-1, -2, and -3-DS by 41%, 45%, and 53%, respectively. Additionally, we found that the theanine content in the pTRV2-*CsWRKY50* lines was higher than that in the control lines after drought stress. This indicates that theanine synthesis in the pTRV2-*CsWRKY50* lines is less affected by drought stress, and the theanine synthesis in these lines is mainly influenced by the expression level of *CsWRKY50* (Fig. 6I). Furthermore, RT-qPCR results showed that the expression of *CsWRKY50* was upregulated and the expression levels of *CsREM1* and *CsTSⅠ* were downregulated in the WT-DS, pTRV2-DS, and pTRV2-*CsWRKY50*-1, 2, 3-DS lines (Fig. 6, J to L). Relative to the pTRV2-*CsWRKY50*-1, 2, 3 lines that were not subjected to drought treatment, we found significant upregulation of *CsWRKY50* in pTRV2-*CsWRKY50*-1, 2, 3-DS lines (Fig. 6M), while the expression of *CsREM1* and *CsTSⅠ* was significantly downregulated (Fig. 6, N and O).

In summary, our data preliminarily suggest that *CsWRKY50* is a negative regulator of theanine synthesis. We also found that CsWRKY50 negatively regulates the transcription of *CsREM1* and downregulates its expression. In general, the downregulation of *CsREM1* expression leads to the inhibition of *CsTSⅠ* transcription and ultimately inhibits theanine synthesis.

### CsWRKY50 binds to the *CsREM1* promoter and inhibits its transcription

**Figure 7.**
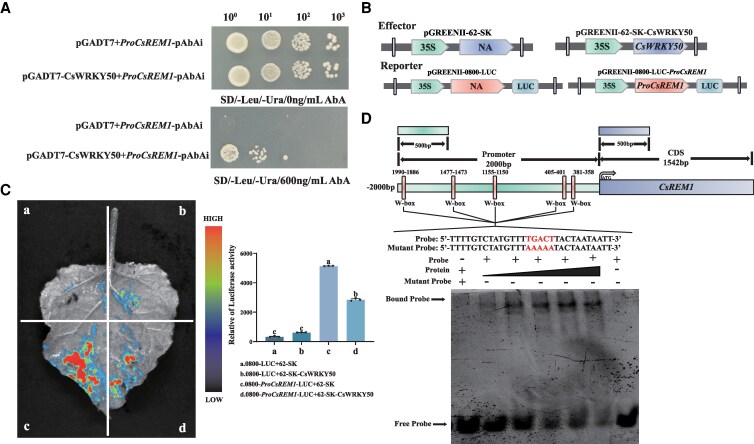
CsWRKY50 binds to the *CsREM1* promoter and negatively regulates *CsREM1* expression. **A)** A Y1H experiment shows interactions between CsWRKY50 and the *CsREM1* promoter. **B)** Structure of the reporter and effector used in the dual luciferase assay. **C)** A dual luciferase expression assay showed that CsWRKY50 enhanced the expression of *CsREM1*. **D)** An EMSA revealed that the CsWRKY50-GST fusion protein bound to W-box motif present in the *CsREM1* promoter region. Error bars represent the mean ± SE of 3 biological replicate experiments. SE, standard error of the mean. Values in **C)** represent the mean ± SE of 3 biological replicates (*n* = 3). Statistical significance of differences among group means was determined using ANOVA and Tukey's HSD tests. Different lowercase letters indicate that there are statistically significant differences between sample means (*P* < 0.05). SD, synthetic dextrose minimal medium; ABA, Aureobasidin A; SD/-Leu/-Ura, SD medium lacking leucine and uracil; SD/-Leu/-Ura/AbA, SD medium containing ABA but lacking leucine and uracil, with the number before ABA representing the concentration of ABA.

Next, we further verified that CsWRKY50 transcriptionally regulates *CsREM1* by, first performing a Y1H. The results of this self-activation assay showed that the self-activation of *ProCsREM1*-pAbAi could be inhibited when the concentration of AbA reached 600 ng/mL (Supplementary Fig. S4B). Moreover, yeast containing pGADT7-*CsWRKY50* and *ProCsREM1*-pAbAi demonstrated robust growth on SD/-Leu/-Ura and SD/-Leu/-Ura/600 ng/mL AbA plates, thereby suggesting that CsWRKY50 can interact with the promoter region of *CsREM1* in vivo (Fig. 7A).

Next, we carried out a dual luciferase expression assay experiment. To do so, the CDS sequence of *CsWRKY50* was inserted into a pGREENII 62-SK vector, and the promoter sequence of *CsREM1* was inserted into a pGREENII 0800-LUC vector (Fig. 7B). A transient expression experiment was then conducted on the leaves of *N. benthamiana*. These results indicate that the relative luciferase activity of *ProCsREM1*::LUC alone was 2 times greater than that when coexpressed with 35S::*CsWRKY50* in tobacco leaves (Fig. 7C).

Finally, to determine the location of the CsWRKY50 binging site in the promoter region of *CsREM1*, we conducted an EMSA. Briefly, the CsWRKY50-GST protein (Supplementary Fig. S5, C and D) was incubated with *CsREM1* promoter sequences of different DNA fragments (i.e. probes) that were then subjected to EMSA analysis. These results showed that CsWRKY50 binds to the TGACT(W-box) DNA sequence motif that is present in the *CsREM1* promoter region (Fig. 7D). In summary, our experimental data indicate that CsWRKY50 functions as a critical TF for negatively regulating theanine content in tea plant leaves, which aligns with findings from WGCNA.

## Discussion

Drought stress serves as a significant environmental regulatory factor influencing both the growth of tea plants and their ability to synthesize secondary metabolites. Recent scientific work has given more attention to the impact of drought stress on the normal growth of tea plants, leading to a greater focus on investigating mechanisms underlying drought resistance in these plants. In general, tea plant leaves are the main source of raw tea, and drought stress can cause leaf damage and loss of secondary metabolites, both of which greatly affect tea quality (Wang et al. 2016a, 2016b; Pan et al. 2017; Chiu et al. 2021; Hoffmann et al. 2023; Cao et al. 2024; Wen et al. 2024). Moreover, the influence of drought stress on secondary metabolites is strong, particularly regarding theanine, which is the primary contributor to the umami flavor present in tea. However, to date, few reports have specifically studied the impact of drought stress on theanine production.

Theanine synthesis is known to be regulated by the key enzyme CsTSI (Sakato 1950; Sasaoka et al. 1963, 1965; Deng et al. 2009; Zhu et al. 2021; Cao et al. 2024; Chen et al. 2024a, 2024b). However, to date, the molecular mechanisms underlying the regulation of theanine synthesis in tea plants under drought stress remain unclear. To explore the molecular mechanisms responsible for this regulatory activity, we simulated drought stress conditions using exogenous treatment with PEG-6000 (Chiu et al. 2021; Jin et al. 2021; Cao et al. 2024) and observed a significant reduction in theanine content as the duration of drought increases and also found that the expression levels of *CsTSI* were strongly downregulated (Fig. 1). This observation is consistent with those of earlier studies that examined the transcriptomic response of tea plants to drought stress (Wang et al. 2016a, 2016b; Zhang et al. 2017). However, the mechanisms responsible for reductions in theanine content and the downregulation of *CsTSI* expression in tea plants subjected to drought stress also unclear. Specifically, it remains uncertain whether TFs or other regulatory elements play a role in this process. Therefore, to investigate this molecular mechanism in greater depth, we first used the *CsTSI* promoter as a bait and performed Y1H and transcriptome analyses to identify the TF CsREM1 from a yeast cDNA library derived from tea plants (Supplementary Fig. S2). Subsequent bioinformatics analysis indicated that CsREM1, which can directly bind to the *CsTSI* promoter, is classified within the REM subfamily of the B3 gene superfamily (Fig. 2 and Supplementary Fig. S3).

In general, the B3 gene family has been found to play key roles in plant embryonic development and seed maturation (Swaminathan et al. 2008), while the REM subfamily is known to be particularly important for hypocotyl and cotyledon morphogenesis (Verma et al. 2022). Like other genes, the B3 family has been most extensively examined in *Arabidopsis* (Matias-Hernandez et al. 2010; Mendes et al. 2016; Manrique et al. 2023) as well as in rice (Wu et al. 2021). However, to the best of our knowledge, to date, no reports have yet examined whether theanine synthesis is regulated by B3 gene family members in tea plants. In this study, we observed a positive correlation between *CsREM1* expression and theanine content in the leaves of tea plants. Using downregulation via VIGS silencing and transient overexpression of *CsREM1*, we generated data that indicates that *CsREM1* positively regulates theanine synthesis. Moreover, in pTRV2-*CsREM1* lines, we observed a reduction in theanine content, which was accompanied by a significant downregulation of *CsTSI* expression. In OE-*CsREM1* lines, we observed a notable increase in theanine content that was accompanied by a significant upregulation of *CsTSI* expression. Taken together, these findings align with the outcomes observed in our VIGS experiment and can help us better understand how TFs regulate theanine synthesis in response to drought conditions. Interestingly, in the absence of drought stress, our data show that CsREM1 upregulates *CsTSI* transcription and promotes theanine synthesis (Fig. 4). However, under drought stress, both theanine content and expression levels of *CsREM1* and *CsTSI* decrease. This observation leads us to speculate whether a TF upstream of *CsREM1* could respond to drought by regulating *CsREM1* transcription, thereby affecting its positive regulation of *CsTSI*. Accordingly, we conducted a comprehensive analysis of the regulatory network involving TFs and employed WGCNA on the drought transcriptome. This analysis led to the identification of the TF CsWRKY50, which exhibits a strong correlation with theanine content, as well as with the abundance of *CsREM1* and *CsTSI*, indicating that it may play a regulatory role (Fig. 5 and Supplementary Fig. S3).

WRKY TFs represent a significant and unique class of TFs in plant biology. Numerous studies have demonstrated that these TFs exert a clear to drought conditions (Chen et al. 2017; Wang et al. 2018; Ma et al. 2019). Our investigation into the drought transcriptome, further supported by RT-qPCR analysis, revealed that *CsWRKY50* is upregulated under drought conditions (Fig. 5). Interestingly, this expression pattern resembles that of *MdWRKY50*, observed in apple (Bai et al. 2024), as well as that of *CsWRKY2* in tea plants (Wang et al. 2016a, 2016b). We subsequently established that CsWRKY50 functions as a TF that is induced by drought and plays a regulatory role in theanine synthesis. Furthermore, the silencing of *CsWRKY50* resulted in a marked increase in theanine levels and an upregulation of the expression of *CsREM1* and *CsTSⅠ* in several pTRV2-*CsWRKY50* lines. Moreover, when these silenced lines were subjected to drought stress, we noted a significant upregulation in *CsWRKY50* expression, while both *CsREM1* and *CsTSⅠ* exhibited significant downregulation, leading to an overall reduction in theanine content. However, in the OE-C*sWRKY50* lines, we observed a notable decrease in theanine levels, accompanied by lowered *CsREM1* and *CsTSⅠ* expression. Taken together, these results confirm that drought stress activates CsWRKY50, which in turn downregulates a positive regulatory pathway involving *CsREM1* and *CsTSⅠ*, which ultimately reduces theanine levels in drought-afflicted tea plants (Fig. 5).

TFs have been found to be involved in the regulatory network that governs the synthesis of theanine. Moreover, they do so via influencing the activity of gene promoters within the theanine synthetic pathway (Chang et al. 2024; Chen et al. 2024a, 2024b; Gao et al. 2025; Mao et al. 2025). CsWRKY50, a member of the WRKY TF family (Teng et al. 2021), is thought to regulate *CsREM1* by specifically interacting with specific DNA sequence motifs such as (T)TGAC(C/T) (W-box), which is located within the *CsREM1* promoter. This interaction was substantiated through a series of experiments, including Y1H, luciferase assays, and EMSA. The results of these experiments collectively established that CsWRKY50 binds to the W-box in the *CsREM1* promoter, thereby inhibiting its transcription (Fig. 7). Moreover, while CsREM1 was identified using Y1H experiments, both Y1H and luciferase expression assay methods are methodologically limited to confirmation direct binding of CsREM1 to the *CsTSⅠ* promoter and cannot further elucidate the specific DNA sequence motifs involved. Furthermore, no studies have yet identified the specific DNA sequence motifs within target gene promoters that are recognized by B3 TFs in tea plants. However, using AtREM1 (in *Arabidopsis*) as a reference point, we performed DNA sequence motif prediction using the JASPAR database (Lin et al. 2024). This analysis led to the identification of 2 distinct DNA sequence motifs: CTACACA and TATGTAG. We then conducted EMSA tests on each of these motifs individually and found that CsREM1 is capable of binding to both sequence motifs identified. Moreover, in combination with the luciferase assay, we found that CsREM1 can directly bind to the promoter of *CsTSⅠ* and upregulate its transcription. Previous studies have substantiated the existence of a regulatory cascade involving the TFs CsWRKY50 and CsREM1 in theanine synthesis under drought stress conditions. Moreover, many studies have elucidated a wide variety of cascading regulatory mechanisms associated with TFs. In 1 previous study, the CsSPX3-*CsPHL7*-*CsTSⅠ*/*CsGS1* module was found to reveal the regulatory mechanism underlying how theanine synthesis can be inhibited in tea plants in response to low phosphorus stress (Chen et al. 2024a, 2024b). In general, the CsREV-*CsTCP4*-*CsVND7* module can be employed in a differential manner to influence the xylem architecture in both leaves and stems and is thought to thereby improve the drought resilience of tea plants (Li et al. 2024). Although numerous studies have reported on cascade regulatory mechanisms involving TFs in tea plants, most have primarily focus on protein–protein interactions between upstream genes and TFs. While such interactions play a crucial role in influencing the regulation of downstream target genes by specific TFs, to date, no reports have yet indicated that upstream TFs can be induced by environmental factors to modulate the activity of downstream TF promoters. This process consequently alters the original regulatory mechanisms employed by downstream TFs to affect the expression levels of target genes. Moreover, whether CsWRKY50 has its own regulatory mechanism of protein interaction with CsREM1, or like CsWRKY2 (Wang et al. 2016a, 2016b), can be regulated by both drought and cold stress and participate in other cascade regulatory networks, remain unknown. We believe that these questions are significant priorities for our future research.

**Figure 8.**
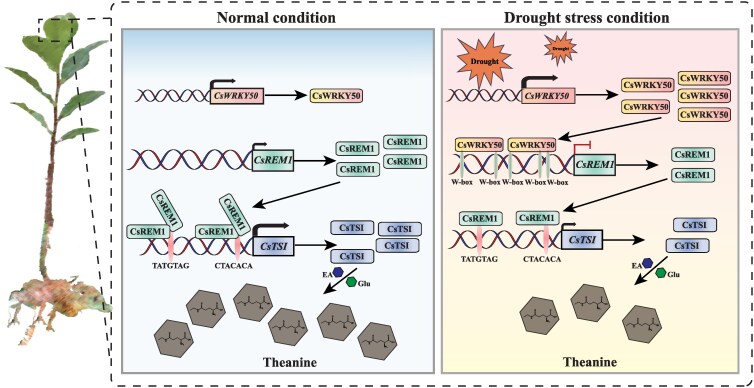
Proposed regulatory model for a CsWRKY50-*CsREM1*-*CsTSⅠ* module that reduces theanine synthesis in tea plant leaves under drought stress. Under normal conditions, CsREM1 binds to the *CsTSI* promoter and positively regulates its transcription. Under drought stress, CsWRKY50 is activated, upregulates its expression, and binds to the *CsREM1* promoter, reducing *CsREM1* transcription. This disrupts CsREM1's positive effect on *CsTSI*, inhibiting *CsTSI* transcription and suppressing theanine synthesis.

In this research, we examined the molecular regulatory mechanisms underlying the synthesis of theanine in tea plants subjected to drought stress. Notably, we report the critical involvement of the CsWRKY50-*CsREM1*-*CsTSI* module in the inhibition of theanine synthesis under drought stress (Fig. 8). Our findings indicate that drought stress significantly diminishes the synthesis of theanine, which correlates with the downregulation of *CsTSI* expression. Subsequent investigations revealed that drought-induced CsWRKY50 inhibits the expression of *CsREM1* by interacting with its promoter. This interaction disrupts the positive regulatory influence of CsREM1 on *CsTSI*, thereby establishing a cascade regulatory network that ultimately results in the decreased synthesis of theanine. This discovery offers a molecular perspective and theoretical framework for comprehending the alterations in theanine levels in tea plants under drought stress.

## Materials and methods

### Plant materials and sample treatment methods

Seedlings (2-yr-old) of the tea cultivar “Fuding dabaicha” were used as experimental materials for all experiments involving tea plants. This particular cultivar was sourced from a tea plantation located at 26° 24′ N, 106° 11′ E (average altitude: 1132 m) that belongs to Guizhou University in Guiyang (Guizhou Province, China). In addition, we used leaves of *N. benthamiana* seedlings as material for dual luciferase expression assay and subcellular localization. All seedlings were cultivated in the greenhouse of Tea College at Guizhou University. Cultivation occurred under controlled conditions (i.e. 25 °C during a 13 h day and 18 °C during an 11 h night). Finally, fresh samples were analyzed directly after harvest, or were flash-frozen in liquid nitrogen, and stored in a freezer at −80 ℃ for further use.

### PEG-6000 was used to simulate drought stress

To simulate drought stress, we used first-year tea seedlings of “Fuding dabaicha” as the experimental material. First, tea seedlings were thoroughly washed twice with clean water and then cultivated in a hydroponic nutrient solution specifically designed for tea seedlings. The composition of this nutrient solution is detailed in Supplementary Table S1. The greenhouse conditions were maintained as above (i.e. 25 °C during a 13 h day and 18 °C during an 11 h night). After a 15 d acclimatization period, tea seedlings were subjected to drought stress by placing them in a hydroponic nutrient solution containing 10% PEG-6000 (Kermel, Tianjin, China). During the drought stress treatment, samples (leaf material) were collected at the following time points: 0, 6, 12, 24, and 48 h. At each time point, 3 individual plants were pooled together to form 1 biological replicate. This procedure was repeated to obtain a total of 3 biological replicates. As described above, harvested samples were promptly placed in liquid nitrogen and stored at −80 °C for subsequent analysis.

### Quantification of theanine content via HPLC

The extraction and quantification of theanine were performed as per the method of GB/T23193-2017 (China) and Huang et al. (2022) with slight modification. Briefly, we first weighed 1.0 g of ground sample and placed it in a 200 ml container. Next, we added 100 ml of boiling ddH_2_O and placed the mixture in a water bath at 100 °C for a 30 min extraction. The extract was then filtered and the filtrate was transferred to a new 100 ml container. Next, samples were diluted with ddH_2_O and mixed well. The filtrate was then pipetted and refiltered through a 0.22 *µ*m hydrophilic membrane. We then transferred the filtrate to a sample vial and stored it at −20 °C for further analysis (or proceeded to immediate analysis, as needed). HPLC conditions were as follows: normal phase C18 column (5.0 *μ*m, 250 mm × 4.6 mm) (NanoChrom, Suzhou, China), column temperature of 35 °C, detection wavelength of 210 nm, ultrapure water used as Mobile Phase A, and 100% acetonitrile (M157, Mreda, Beijing, China) as Mobile Phase B. HPLC was run using an extract volume of 10 *µ*l.

### Transcriptome sequencing, total RNA extraction, and RT-qPCR analysis

Transcriptome sequencing of drought stress samples from each of the 5 time periods in the drought treatment was performed using an Illumina Nova Seq 6000 platform (Beijing, China) at Novagene. Sequencing of each sample was repeated 3 times. First, total RNA was extracted from frozen samples using a modified cetyltrimethylammonium bromide (CTAB) method (Lu et al. 2021). Next, RT-qPCR assays were performed using Golden Star RT6 cDNA Synthesis Kit (TSK302M, Tsingke, Beijing, China), ChamQ Blue Universal SYBR qPCR Master Mix (Q312-02, Vazyme, Nanjing, China), and a Bio-Rad CFX Connect real-time quantitative PCR platform. The actin (CSS0008920) gene present in tea plants was used as the reference gene for expression normalization. Gene expression quantification during RT-qPCR was performed using the SYBR Green method. RT-qPCR was performed under the following cycling conditions: 95 °C for 30 s, followed by 40 cycles of 95 °C for 10 s and 60 °C for 30 s, and a final dissociation curve of 65 °C for 0.05 s and 95 °C for 0.5 s. After PCR, the relative expression levels of target genes were assessed using the 2^−ΔΔCt^ method. All primer sequences used for expression quantification are listed in Supplementary Table S3.

### Subcellular localization

For subcellular localization experiments, full-length *CsREM1* and *CsWRKY50* sequences were first cloned from tea plant leaf cDNA and inserted into a GFP expression pCAMBIA1300 vector to obtain fusion constructs 35S::*CsREM1*-GFP and 35S::*CsWRKY50*-GFP. Both were subsequently transformed into *A. tumefaciens* GV3101, and the empty vector pCAMBIA1300-GFP was used as a negative control. p2300-35S-H2B-mCherry was used as a nuclear localization marker. Next, the *Agrobacterium* suspension was slowly injected into the leaves of *N. benthamiana*. Subcellular localization was performed 48 h after injection with *Agrobacterium* suspension. The injected tobacco leaves were sectioned, and fluorescence signals were observed according to the instructions for the ZEISS LSM900 laser confocal microscope (excitation light source, GFP 488 nm and mCherry/RFP 560 to 610 nm; intensity, 5% to 20%; collection bandwidth, GFP 500 to 550 nm and mCherry/RFP 580 to 650 nm; gain, 500 to 700). All primers used are listed in Supplementary Table S3.

### Y1H assay

Tea leaf cDNA yeast libraries were constructed using a Yeast Hybrid Library Construction Kit (JKR23009H, GENE CREATE, Wuhan, China). Firstly, we cloned the *CsREM1* and *CsTSⅠ* promoters from the tea plant genomic DNA and created the *ProCsTSⅠ*-pAbAi and *ProREM1*-pAbAi vectors. Next, the coding sequences (CDSs) of *CsREM1* and *CsWRKY50* were cloned into pGADT7 to produce the pGADT7-*CsWRKY50* and pGADT7-*CsREM1* constructs.

We then digested the *ProCsTSⅠ*-pAbAi recombinant plasmid with BstBI and then cotransformed it with pGADT7 into Y1H competent cells (CC308, Coolaber, Beijing, China). Transformed yeast cells were then selected on a SD/-Leu (PM2201, Coolaber, Beijing, China) selection plate containing 0, 100, 300, and 600 ng/mL Aureobasidin (AbA) (CA2332, Coolaber, Beijing, China) to screen for inhibition of self-activation. In addition, the pGADT7-*CsREM1* plasmid was cotransformed with *ProCsTSⅠ*-pAbAi plasmids into Y1H competent cells and then plated on the SD/-Leu/-Ura (PM2291, Coolaber, Beijing, China) medium containing AbA for a 3 d incubation. Since the promoter sequence of *ProCsREM1*-pAbAi contains the restriction site sequence for BstBI, BsbI was used for linearization. The rest of the experimental method was consistent with that reported above for *ProCsTSⅠ*-pAbAi.

### Dual luciferase expression

For dual luciferase expression, the full-length CDSs of *CsREM1* and *CsWRKY50* were first cloned and inserted into a pGREENII 62-SK expression vector. Next, the promoter sequences of *CsREM1* and *CsTSⅠ* were cloned and inserted into the pGREENII 0800-Luc expression vector. This fusion vector was then transformed into GV3101 (pSoup) competent cells (Bio-Transduction Lab, Wuhan, China). After injection with the vector-containing cells, *N. benthamiana* leaves were cultured in a dark environment for 3 to 5 d. After recovery, tobacco leaves were sprayed with 0.2 mg·mL^−1^ D-luciferin potassium (IL2330, Solarbio, Beijing, China) and then incubated at room temperature for 10 min. A Fusion X7 chemiluminescence imaging system (VILBER, France) was used for fluorescence imaging. All primers used in these procedures are listed in Supplementary Table S3

### EMSA

For EMSA, the CDS sequences of *CsWRKY50* and CsREM1 were first inserted into pGEX-4T-GST and transformed into Rosetta strain *E. coli* cells (Bio-Transduction Lab, Wuhan, China). Monoclonal colonies were then selected and cultured in liquid LB medium; here, both liquid and solid media contained 100 mg/L ampicillin and 34 mg/L chloramphenicol. When the OD_600_ reached 0.5 to 0.8, we added isopropyl β-D-thiogalactopyranoside (IPTG) (I8070, Solarbio, Beijing, China) at a working concentration of 1 mm to induce protein expression. The solution was then kept at 28 °C for 12 to 16 h. After protein expression, the induced recombinant protein was purified using a GST Sep Glutathione Agarose Resin (20507ES50, Yeasen, Shanghai, China) and glutathione, reduced (GSH) (60342ES10, Yeasen, Shanghai, China). Finally, we performed EMSA using a protocol originally established by Zhu et al. (2024). The sequences of all probes used in this experiment are provided in Supplementary Table S3.

### VIGS of tea plants

For gene silencing experiments, we targeted the *CsREM1* and *CsWRKY50* genes in the tea cultivar “Fuding dabaicha” for silencing using VIGS (Li et al. 2023). To do so, fragments of *CsREM1* and Cs*WKY50* suitable for VIGS were first inserted into the TRV2 viral vector to construct pTRV2-*CsREM1* and pTRV2-*CsWRKY50* vectors. Next, pTRV1, pTRV2, pTRV2-*CsREM1*, and pTRV2-*CsWRKY50* were individually transformed into *Agrobacterium* strain GV3101 chemically competent cells (Bio-Transduction Lab, Wuhan, China). The *Agrobacterium* solution containing pTRV1 was resuspended with an *Agrobacterium* solution carrying pTRV2, pTRV2-*CsREM1*, and pTRV2-*CsWRKY50* at a ratio of 1:1 (v:v). Next, pruned tea cuttings underwent vacuum impregnation before being cultured in a dark environment for 3 d. After recovery, plants were transferred to a greenhouse kept at 25 °C for a culture cycle of 16 h light/8 h dark. Samples were collected at the 35 d time point. During sampling, 3 biological replicates were grouped together, resulting in a total of 3 distinct groups. Samples were immediately frozen in liquid nitrogen and stored in a −80 °C freezer. All primers used are listed in Supplementary Table S3.

### Transient overexpression in tea plant leaves

Transient expression in tea plant leaves was performed as previously reported by Li et al. (2022). The same vector was used as for subcellular localization experiments (see above). Here positive monoclonal strains were cultured in liquid YEP medium containing 50 mg/L rifampicin (R8011, Solarbio, Beijing, China) and 50 mg/L kanamycin (K8020, Solarbio, Beijing, China). When the required OD value was reached, it was suspended in a suspension buffer containing 10 mm MES (M8010, Solarbio, Beijing, China) (pH 5.6), 10 mm MgCl_2_ (M8161, Solarbio, Beijing, China), and 200 *μ*M acetosyringone (AS) (A8110, Solarbio, Beijing, China), until it reached a final OD_600_ of 1.0. At this point, tea plant leaves were divided into 2 parts along the main vein. An *Agrobacterium* suspension containing the pCAMBIA2301-35S-GFP vector was injected on the left side as a control, and a suspension containing the inserted gene was injected on the right. Samples were collected 5 d after injection and analyzed. For each combination, 3 biological replicates were grouped together, resulting in a total of 3 distinct groups. At harvest, all samples were immediately frozen in liquid nitrogen and stored in a −80 °C freezer until further use. All primers used are listed in Supplementary Table S3.

### Construction of coexpression networks and subsequent WGCNA

WGCNA was conducted using the WGCNA R package (Langfelder and Horvath 2008), according to a previously described protocol (Qiao et al. 2023; Tian et al. 2024), with some modifications. Briefly, differentially expressed genes (DEGs) were identified based on the following conditions: a single sample gene expression level of FPKM > 10 and a coefficient of variation (CV) > 0.5 in 15 samples. These DEGs served as the input for the WGCNA analysis. The primary parameters were configured as follows: power = 30, minimum module size = 30, and merge cut height = 0.2. All other parameters were set to their default settings. Finally, Cytoscape version 3.10.0 was used to visualize the module network.

### Bioinformatics analysis

Conserved domain analysis was conducted using the CD-Search function as implemented by the NCBI database. Chromosome location analysis was performed in TBtools (Chen et al. 2023) using the “shuchazao_V2” genome file and “shuchazao_V2” gff3 file sourced from the TPIA database. The amino acid sequences of proteins subjected to phylogenetic analysis and tree construction were aligned using the Align by ClustalW function as implemented in MEGA version 11.0. Finally, we constructed a phylogenetic tree using the Construct/Test Neighbor-Joining Tree function, also implemented in MEGA version 11.0 (i.e. bootstrap = 1000).

### Statistical analyses

All assays were performed using 3 biological replicates and analyzed using SPSS version 27.0 (IBM SPSS, Armonk, USA). All experimental data were obtained from at least 3 independent experiments with 3 biological replicates. Pearson's correlation analysis was used to analyze experimental data produced during this study. Finally, Student's *t* test, 1-way ANOVA, and Tukey's Honestly Significant Difference (Tukey's HSD) tests were used to assess the statistical significance of differences in group mean values.

### Accession numbers

Sequence data from this article can be found in the GenBank/EMBL data libraries under accession numbers CsTSI(DD410895.1), CsREM1 (XM_028246078.1), and CsWRKY50 (XM_028206267.1).

In addition to the accession numbers mentioned above, specific sequence information can be found in the preceding text, Supplementary Tables S4 and S5, as well as their accession numbers in the TPIA database and sequence date.

## Supplementary Material

kiaf437_Supplementary_Data

## Data Availability

The relevant research data for this study are provided in the supplementary materials. If additional data involving laboratory core secrets are required, please contact the authors of this paper.

## Supplementary data

The following materials are available in the online version of this article.

**Supplementary Figure S1.** Pearson's correlation analysis was used to analyze the correlation between theanine content and the related genes of the theanine synthesis pathway under drought stress.

**Supplementary Figure S2.** Screening of TFs binding to the *CsTSI* promoter region by Y1H.

**Supplementary Figure S3.** Chromosomal localization and phylogenetic tree analysis of CsREM1 (CSS0032554) and CsWRKY50 (CSS0032848).

**Supplementary Figure S4.**  *ProCsTSI*-pAbAi(A) and *ProCsREM1*-pAbAi(B) self-activation assay and AbA concentration screening.

**Supplementary Figure S5.** The proteins of CsREM1-GST and CsWRKY50-GST were successfully expressed and extracted.

**Supplementary Table S1.** Hydroponic nutrient solution formulation table for tea plants.

**Supplementary Table S2.** The expression profile of genes related to the theanine synthesis pathway under drought stress.

**Supplementary Table S3.** Primers and probes were used in this study.

**Supplementary Table S4.** The CDS sequences and amino acid sequences of *CsREM1* and *CsWRKY50*.

**Supplementary Table S5.** The promoter sequences of *CsTSI* and *CsREM1*.

## Dive Curated Terms

The following phenotypic, genotypic, and functional terms are of significance to the work described in this paper:

MEGA CHEBI: CHEBI:6617

NCBI Gramene: https://www.ncbi.nlm.nih.gov
NCBI Araport: https://www.ncbi.nlm.nih.gov

JASPAR Gramene: https://jaspar.elixir.no/
JASPAR Araport: https://jaspar.elixir.no/
