# Supplementary material for: The CsWRKY50-CsREM1-CsTSⅠ module inhibits theanine biosynthesis in tea plants under drought stress
Source: Plant Physiol. 2025 Sep 25;199(2):kiaf437. doi: 10.1093/plphys/kiaf437 (PMC12507091; doi:10.1093/plphys/kiaf437)
Supplement: kiaf437_Supplementary_Data [file kiaf437_supplementary_data.pdf]

The CsWRKY50-*CsREM1*-*CsTSI* module inhibits theanine biosynthesis in tea plants under drought stress

Shenyuan Ye[1], Linlin Li[1], Ping Li[1, 2], Xinzhuan Yao[1, 2], Qi Zhao[1, 2], Shiyu Tian[2], Tong Li[1], Yihe Jiang[1], Zhenkedai Yuan[2], Yu Chen[2], Qi-hong Zou[1], Shi-yu Zhang[2], Yue Wan[4], Chao Xu[5], Hui Hu[5], Zifan Yang[4], Chao Luo [1, 3*], Li-Tang Lu[1, 2*]

[1]College of Tea Science and The Key Laboratory of Plant Resources Conservation and Germplasm Innovation in Mountainous Region (Ministry of Education), Guizhou University, Guiyang, 550025, China.

[2]College of Life Science, Guizhou University, Guiyang, 550025, China.

[3]College of Forestry, Guizhou University, Guiyang, 550025, China.

[4]HUANENG CLEAN ENERGY RESEARCH INSTITUTE, Beijing, 102209, China.

[5]HUANENG Guizhou Clean Energy Branch, Guiyang, 550081, China.

*Corresponding author: Chao Luo(chaoluo@gzu.edu.cn); Li-Tang Lu (ltlv@gzu.edu.cn)

Supplementary Table S1. Hydroponic Nutrient Solution Formulation Table for Tea Plants.

Chemical Compound	Compounds Provide Elements	Compound Concentration
$(NH_4)_2SO_4$	N	0.25mM
NH_4NO_3	N	0.5mM
KH_2PO_4	P, K	0.5mM
K_2SO_4	P, K	0.2mM
$MgSO_4 \cdot 7H_2O$	Mg, S	0.8mM
$Al_2(SO_4)_3 \cdot 18H_2O$	Al	0.08mM
$Ca(NO_3)_2 \cdot 4H_2O$	Ca	0.5mM
$FeSO_4 \cdot 7H_2O$	Fe	17.5μM
$Na_2 \cdot EDTA$	Fe	17.5μM
H_3BO_3	B	23μM
$MnSO_4 \cdot 4H_2O$	Mn	4.5μM
$ZnSO_4 \cdot 7H_2O$	Zn	1μM
$CuSO_4 \cdot 5H_2O$	Cu	0.15μM
$Na_2MoO_4 \cdot 2H_2O$	Mo	0.05μM

Note: $Na_2 \cdot EDTA$ was used to promote the dissolution of $FeSO_4 \cdot 7H_2O$.

Supplementary Table S2. The expression profile of genes related to the theanine synthesis pathway under drought stress.

Name	Gene ID	PEG_0h1	PEG_0h2	PEG_0h3	PEG_6h1	PEG_6h2	PEG_6h3	PEG_12h1	PEG_12h2	PEG_12h3	PEG_24h1	PEG_24h2	PEG_24h3	PEG_48h1	PEG_48h2	PEG_48h3
CsTSI	CSS0007224	443.04771	438.49127	455.43006	392.442	397.94365	383.60481	231.21576	227.12132	235.56628	185.10001	191.47871	197.72748	91.289362	87.927645	90.974835
CsTSII	CSS0037306	74.576744	52.452904	118.4213	78.265309	25.242715	51.57999	26.974522	67.386329	60.677894	61.828304	43.546158	57.615735	74.10808	53.813276	49.54607
CsTSIII	CSS0034978	43.508626	46.152945	41.565673	39.554931	60.845775	31.507504	42.855464	45.691539	46.64397	18.919867	26.086361	23.81086	20.474264	29.284028	33.058983
CsTSIV	CSS0049154	20.062709	19.794506	20.102768	20.62855	24.720168	22.383868	66.732645	64.62285	66.566295	103.2341	102.78338	98.015379	65.572921	63.783352	65.440613
CsGS1	CSS0026308	11.553223	9.6273887	10.096906	20.756351	16.3097	17.303861	20.820431	18.057574	20.518294	31.736468	27.314755	27.551779	39.149803	37.740536	41.442885
CsGS2	CSS0015313	5.8376522	12.303761	14.316312	46.300314	46.217553	40.764767	16.665137	25.328362	23.111114	10.090065	19.156449	12.082331	35.775368	30.022097	21.47238
CsGS3	CSS0007310	148.16996	145.85106	150.56988	154.36126	167.10919	163.31071	143.05281	144.03994	141.667	35.447854	40.350349	41.587894	10.436366	12.865139	12.96329
CsGS4	CSS0037306	74.576744	52.452904	118.4213	78.265309	25.242715	51.57999	26.974522	67.386329	60.677894	61.828304	43.546158	57.615735	74.10808	53.813276	49.54607
CsGS5	CSS0034978	43.508626	46.152945	41.565673	39.554931	60.845775	31.507504	42.855464	45.691539	46.64397	18.919867	26.086361	23.81086	20.474264	29.284028	33.058983
CsGS6	CSS0049154	20.062709	19.794506	20.102768	20.62855	24.720168	22.383868	66.732645	64.62285	66.566295	103.2341	102.78338	98.015379	65.572921	63.783352	65.440613
CsGOGAT1	CSS0044676	122.86985	127.1265	123.35735	115.98898	116.61409	111.85896	110.25123	109.47194	106.3601	50.990257	48.018784	48.818706	15.626275	14.652362	14.132786
CsGOGAT2	CSS0050330	81.754683	81.851218	81.57357	51.107856	49.003703	47.513715	81.66071	75.127088	75.794974	110.14022	103.17149	99.657953	174.42328	157.70982	164.97365
CsGOGAT3	CSS0007758	62.316597	58.63014	60.460302	60.456254	59.249093	59.188233	61.466268	57.562008	58.226698	46.82886	43.646804	45.210518	90.944976	85.43344	88.195328
CsGOGAT4	CSS0028733	27.162452	29.237573	28.778719	29.925313	30.066738	28.708434	32.298913	31.819954	27.555719	43.307814	39.824826	33.079341	36.309061	38.880876	36.530378
CsGOGAT5	CSS0005230	37.994414	39.135215	39.494676	42.864924	39.257886	41.70373	43.291454	43.3233	41.021053	52.79505	51.133491	45.998388	48.003715	49.795265	50.263051
CsGOGAT6	CSS0050084	35.468738	34.30963	35.507511	16.463685	15.57204	18.493412	17.722216	18.001503	20.39887	7.3906185	6.9721378	5.4358838	5.917078	5.6212976	5.5032695
CsGDH1	CSS0034454	0.3473923	0.3949317	0.4191035	5.6251328	4.4429344	6.6149051	19.973009	20.624212	21.765044	60.664087	61.440288	65.173079	242.90991	244.7929	251.17023
CsGDH2	CSS0007238	11.085036	10.366494	9.7260276	7.1300493	7.6382719	8.3990694	9.364314	9.8215997	9.9505763	17.895643	19.615086	16.608382	22.529544	22.587017	25.629989
CsGDH3	CSS0002543	29.99898	30.909508	32.291647	40.230346	37.46765	39.12782	24.452054	24.257373	22.83872	11.001223	13.10336	10.300507	5.3163206	4.8777169	4.1662605
CsGDH4	CSS0046767	6.1606787	5.9984234	5.689883	15.27372	16.397416	16.39911	6.6771507	5.7309343	6.1314005	2.5137473	2.0660917	1.9548626	3.1769645	3.989031	3.4165446
CsGDH5	CSS0002183	4.6858799	5.796157	5.4386998	8.3280609	7.8965149	8.6241643	6.4456164	5.8190561	5.4807711	4.1762544	5.5049	6.2808876	4.1156951	5.3966115	4.4823598
CsGDH6	CSS0021774	21.939218	23.535014	22.345251	21.348045	21.116633	19.756918	19.798822	19.308434	19.026416	17.193669	16.986489	17.160614	19.314649	18.385419	18.744925
CsGDH7	CSS0036616	8.7378152	7.6454572	8.3956022	6.312845	5.8117968	5.8544505	7.3791195	8.3571762	8.9399063	8.4364152	7.9979236	8.0607131	7.757299	6.1552328	9.2036107
CsALT1	CSS0049060	65.891601	65.817414	70.367652	40.665964	44.263124	42.427972	72.74382	78.383471	72.364456	173.5901	169.72306	173.397	161.98675	171.10584	166.23029
CsAlaDC1	CSS0024054	38.433728	38.3075	38.032118	48.233776	39.648102	40.022101	99.393402	92.765058	102.72707	95.623769	99.941696	90.219491	52.053846	48.849877	50.539367
CsAlaDC2	CSS0028985	63.398489	69.811125	69.800148	109.44892	109.85479	108.42817	171.12748	174.6693	169.51041	167.69339	175.44265	171.31545	103.97166	88.068774	101.465
CsAlaDC3	CSS0040307	3.3630539	2.8757891	3.3953819	6.7589742	6.0108924	5.8667779	14.160406	14.009394	12.196177	44.751613	39.808565	40.19189	36.458124	29.062476	31.438477
CsAlaDC4	CSS0006469	3.0226162	2.4510319	2.3205421	6.4174252	4.9924526	4.6381678	10.98995	9.4834866	10.356024	31.499488	32.991435	29.272537	26.680319	24.631525	22.55367

Note:Those date were from our own lab.

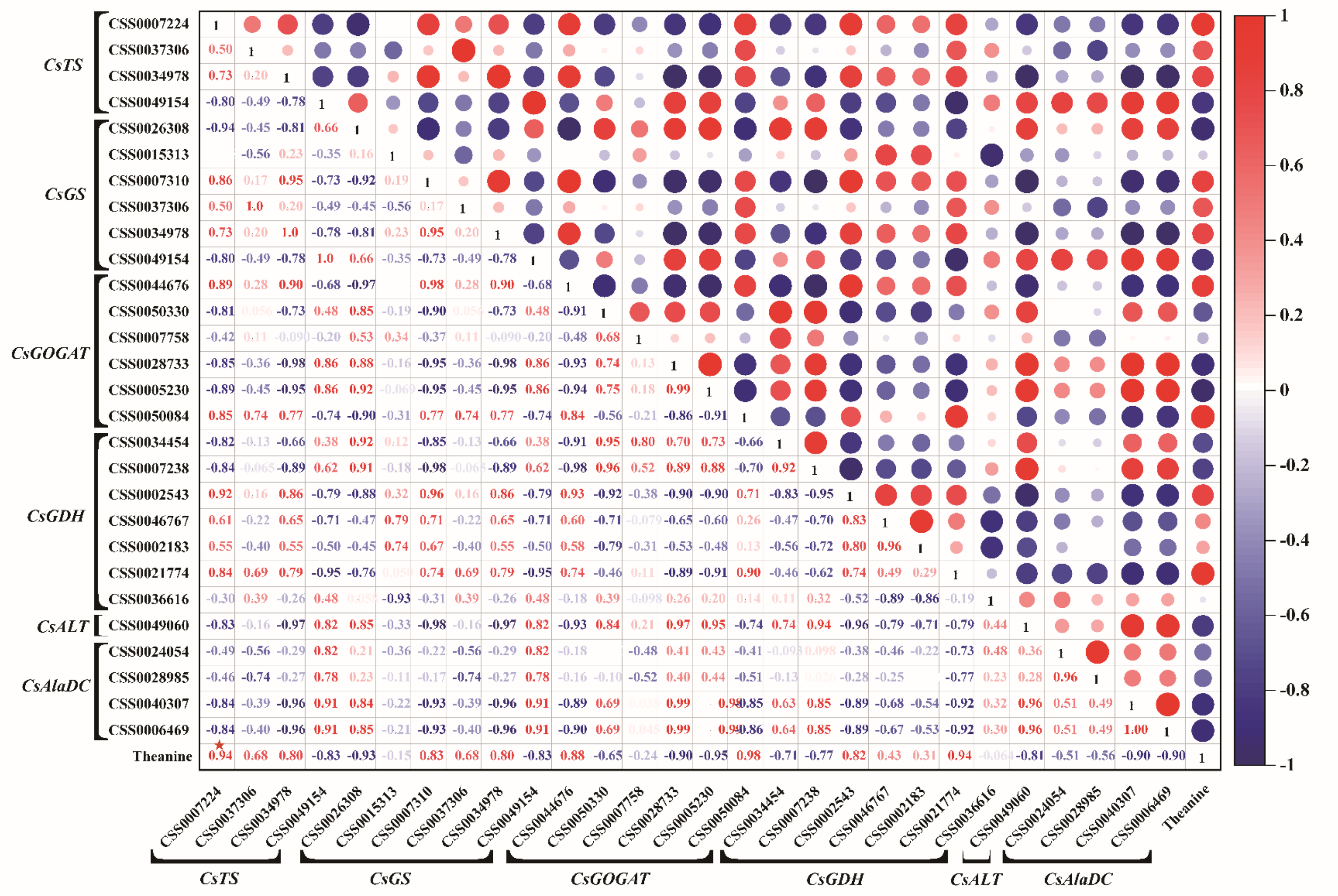

Supplementary Figure S1. Pearson correlation analysis was used to analyze the correlation between theanine content and the related genes of the theanine synthesis pathway under drought stress.

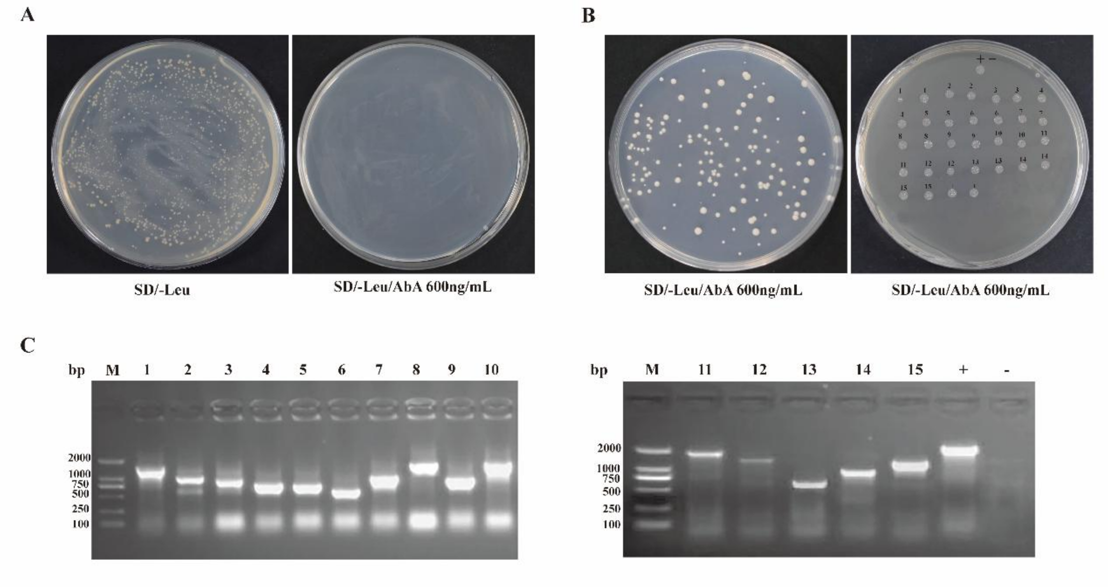

Supplementary Figure S2. Screening of transcription factors binding to *CsTSI* promoter region by Y1H. (A) Verification of self-activation for the bait plasmid (*ProCsTSI*-pAbAi) and assessment of AbA concentration. (B) *ProCsTSI*-pAbAi and pGADT7-library were co-transformed, spread, and spotted onto yeast cells selected by SD/-Leu/AbA 600ng/mL. (C) Lane M: 2000bp DNA Marker; Lane 1-15: PCR results of positive clone; +: positive control; -: negative control; SD, Synthetic Dextrose Minimal Medium; ABA, Aureobasidin A; SD/-Leu, SD medium lacking leucine; SD/-Leu/AbA, SD medium containing ABA but lacking leucine, with the number after ABA representing the concentration of ABA.

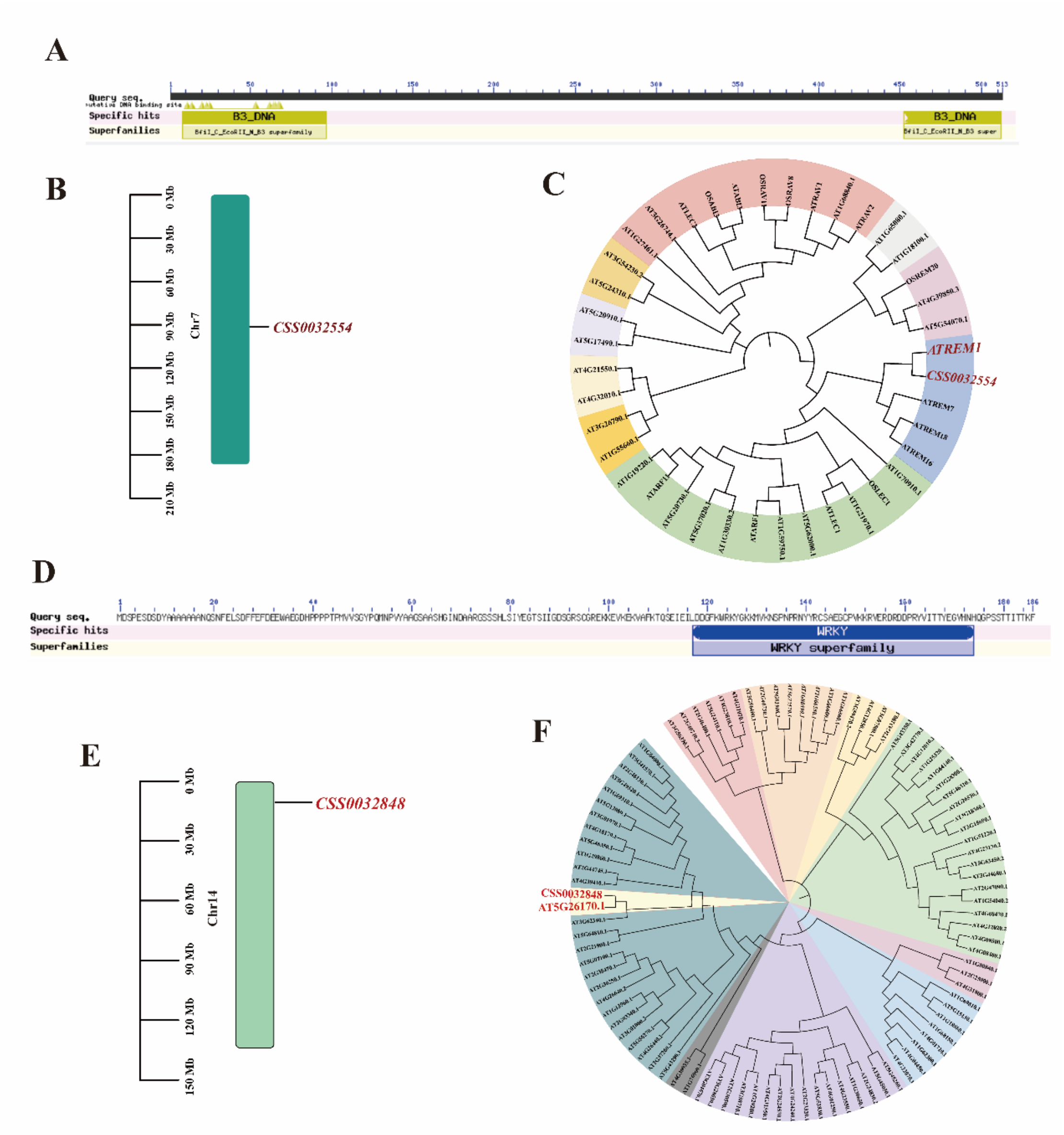

Supplementary Figure S3. Chromosomal localization and phylogenetic tree analysis of CsREM1 (CSS0032554) and CsWRKY50 (CSS0032848).

(A) Conserved domain analysis of CsREM1.

(B) Chromosomal localization of *CsREM1*(CSS0032554).

(C) Phylogenetic analysis of CsREM1 in the reported B3 gene family genes.

(D) Conserved domain analysis of CsWRKY50.

(E) Chromosomal localization of *CsWRKY50*(CSS0032848).

(F) Phylogenetic analysis of CsWRKY50 with Arabidopsis.

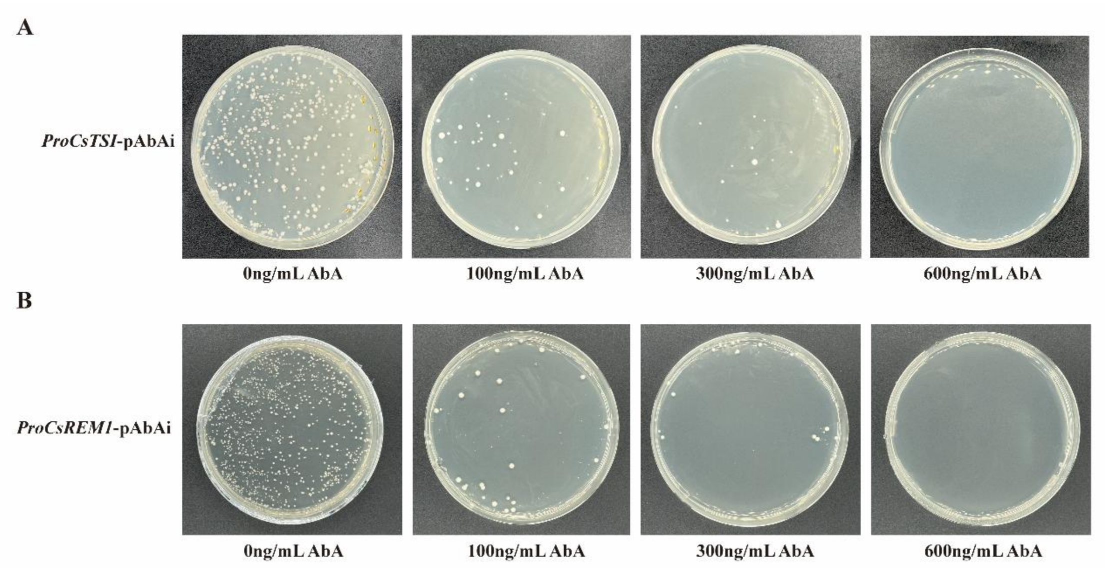

Supplementary Figure S4. ***ProCsTSI*-pAbAi(A) and *ProCsREM1*-pAbAi(B) self-activation assay and AbA concentration screening.**

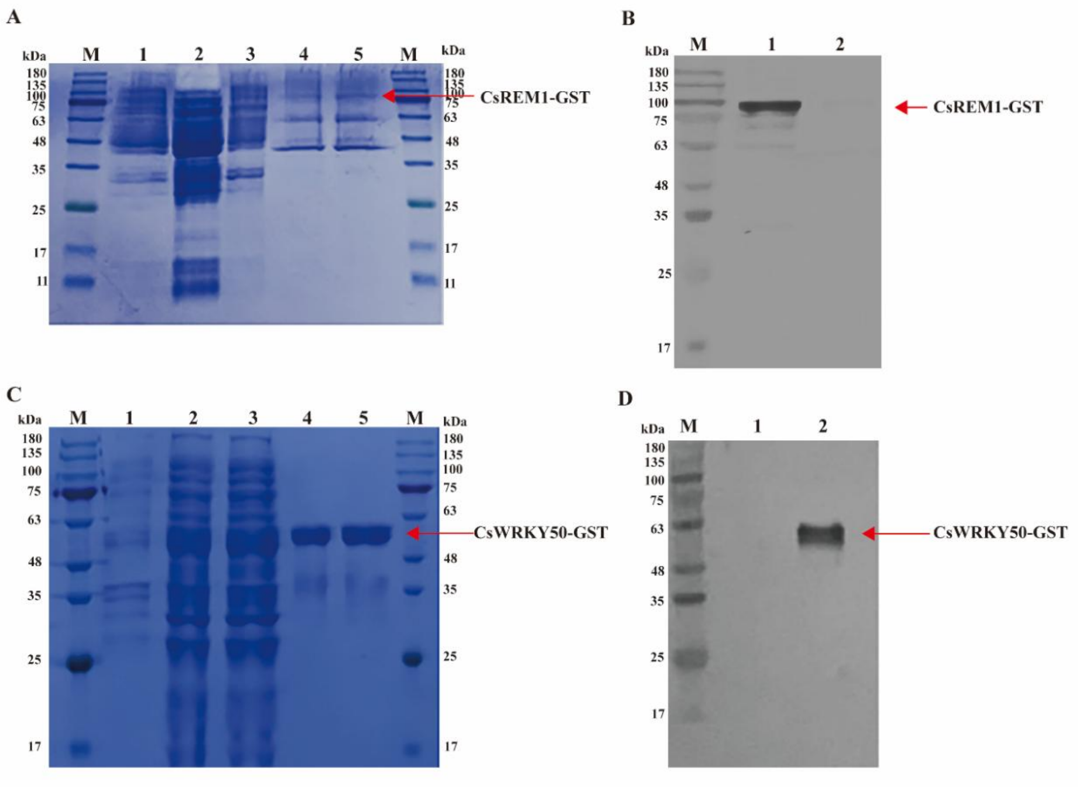

Supplementary Figure S5. The protein of CsREM1-GST and CsWRKY50-GST were successfully expressed and extracted. (A). SDS polyacrylamide gel electrophoresis showed that the CsREM1-GST protein was successfully expressed. M: ColorMixed Protein Marker(11-180KD); 1: not induced; 2: The supernatant was broken after induction; 3: The bacterial precipitation was broken after induction; 4,5: The CsREM1-GST protein was purified by GST Sep Glutathione Agarose Resin. (B). Protein Western Blot identification analysis. M: ColorMixed Protein Marker (11-180KD); 1: not induced; 2: The CsREM1-GST protein was purified by GST Sep Glutathione Agarose Resin. (C). SDS polyacrylamide gel electrophoresis showed that the CsWRKY50-GST protein was successfully expressed. M: ColorMixed Protein Marker(11-180KD); 1: not induced; 2: The supernatant was broken after induction; 3: The bacterial precipitation was broken after induction; 4,5: The CsWRKY50-GST protein was purified by GST Sep Glutathione Agarose Resin. (D)Protein Western Blot identification analysis. M: ColorMixed Protein Marker (11-180KD); 1: not induced; 2: The CsWRKY50-GST protein was purified by GST Sep Glutathione Agarose Resin; SDS, Sodium Dodecyl Sulfate; GST, Glutathione S-Transferase

Supplementary Table S3. Primers and probes were used in this study.

Primer name	Sequence(5'to3')	Function
ProCsREM1-HindIII-F	GATAAGCTTAAACAAGGCCTTAGAAT	Constructing *ProCsREM1*-pGreenll-0800-Luc
ProCsREM1- PstI-R	GGGCTGCAGCTCTTCTCCACTGATGAAATTC	Constructing *ProCsREM1*-pGreenll-0800-Luc
CsREM1-PstI-F	GGGCTGCAGATGGGTTGGCCTGTTGC	Constructing *CsREM1*-pGreenll-62-SK
CsREM1-HindIII-R	GATAAGCTTTTACTTCCTATCAACACTAAC	Constructing *CsREM1*-pGreenll-62-SK
ProCsTSI-HindIII-F	GATAAGCTTAGACACACCTAACATAAATACA	Constructing *ProCsTSI*-pGreenll-0800-Luc
ProCsTSI-PstI-R	GGGCTGCAGGAGTGGGTTTTATAGAGTCCAG	Constructing *ProCsTSI*-pGreenll-0800-Luc
CsWRKY50-PstI-F	GGGCTGCAGATGGATTCTCCGGAATCG	Constructing *CsWRKY50*-pGreenll-62-SK
CsWRKY50-HindIII-R	GATAAGCTTTCAGAACTTGGTAGTAATAG	Constructing *CsWRKY50*-pGreenll-62-SK
CsWRKY50-EcoRI-F	AGTGAATTCATGGATTCTCCGGAATCG	Constructing *CsWRKY50*-pGADT7
CsWRKY50-BamHI-R	GATGGATCCTCAGAACTTGGTAGTAATAG	Constructing *CsWRKY50*-pGADT7
CsREM1-EcoRI-F	AGTGAATTCATGGGTTGGCCTGTTGC	Constructing *CsREM1*-pGADT7
CsREM1-BamHI-R	GATGGATCCTTACTTCCTATCAACACTAAC	Constructing *CsREM1*-pGADT7
CsREM1- SacI-F	TTCGAGCTCAAACAAGGCCTTAGAAT	Constructing *ProCsREM1*-pABAi
CsREM1- KpnI-R	CCGGGTACCCTCTTCTCCACTGATGAAATTC	Constructing *ProCsREM1*-pABAi
CsWRKY50-EcoRI -F	ATCCCCGGAATTCATGGATTCTCCGGAATCG	Constructing *CsWRKY50*-pGEX-4T-GST
CsWRKY50- SalI -R	CTCGAGTCGACTCAGAACTTGGTAGTAATAG	Constructing *CsWRKY50*-pGEX-4T-GST
CsREM1- EcoRI -F	ATCCCCGGAATTCATGGGTTGGCCTGTTGC	Constructing *CsREM1*-pGEX-4T-GST
CsREM1- SalI -R	CTCGAGTCGACTTACTTCCTATCAACACTAAC	Constructing *CsREM1*-pGEX-4T-GST
CsREM1- KpnI-R	GACGGTACCATGGGTTGGCCTGTTGC	Constructing *CsREM1*-pCAMBIA2301-GFP
CsREM1- XbaI-R	GAGTCTAGATTACTTCCTATCAACACTAAC	Constructing *CsREM1*-pCAMBIA2301-GFP

CsWRKY50-KpnI-R	GACGGTACCATGGATTCTCCGGAATCG	Constructing *CsWRKY50*-pCAMBIA2301-GFP
CsWRKY50-XbaI-R	GAGTCTAGATCAGAACTTGGTAGTAATAG	Constructing *CsWRKY50*-pCAMBIA2301-GFP
CsREM1-qPCR-F	CAACAGAAGTTTGCCGATGTG	Measuring the relative expression level of *CsREM1* in qPCR
CsREM1-qPCR-R	CATCTTCCTTGCCCTCATCTC	Measuring the relative expression level of *CsREM1* in qPCR
CsWRKY50-qPCR-F	TACGGCAAGAAGATGGTCAAA	Measuring the relative expression level of *CsWRKY50* in qPCR
CsWRKY50-qPCR-R	TGGACACCCTCGTAAGTAGT	Measuring the relative expression level of *CsWRKY50* in qPCR
CsTSI-qPCR-F	CTTGCCTCTATGCTGGTGTAA	Measuring the relative expression level of *CsTSI* in qPCR
CsTSI-qPCR-R	CAATCTCCGTGATCCTCTCAAG	Measuring the relative expression level of *CsTSI* in qPCR
CsREM1 promoter Probe-F	TTTTGTCTATGTTTTGACTTACTAATAATT	EMSA
CsREM1 promoter Probe-R	AATTATTAGTAAGTCAAAACATAGACAAAA	EMSA
CsREM1 promoter Mutant Probe-R	TTTTGTCTATGTTTAAAAATACTAATAATT	EMSA
CsREM1 promoter Mutant Probe-R	AATTATTAGTATTTTTAAACATAGACAAAA	EMSA
CsTSI promoter Probe-1-F	TATGTAATTTTATGTAGAGTTATACAT	EMSA
CsTSI promoter Probe-1-R	ATGTATAACTCTACATAAAATTACATA	EMSA
CsTSI promoter Probe-2-F	TTACAACATGCTACACAGACTCGTAAA	EMSA
CsTSI promoter Probe-2-R	TTTACGAGTCTGTGTAGCATGTTGTAA	EMSA
CsTSI promoter Mutant Probe-1-F	TATGTAATTTCCCCCCCAGTTATACAT	EMSA
CsTSI promoter Mutant Probe-1-R	ATGTATAACTGGGGGGGAAATTACATA	EMSA
CsTSI promoter Mutant Probe-2-F	TTACAACATGAAAAAAAGACTCGTAAA	EMSA
CsTSI promoter Mutant Probe-2-R	TTTACGAGTCTTTTTTTCATGTTGTAA	EMSA

Supplementary Table S4. The CDS sequences and amino acid sequences of *CsREM1* and *CsWRKY50*.

Name	Gene ID	CDS sequence	The number of nucleotides in the CDS sequence	Amino acid sequence	The number of amino acid in the CDS sequence	Theoretical and actual sizes of the recombinant protein
CsREM1	CSS0032554	ATGGGTTGGCCTGTTGCTACCTCTTTTTTCAAAATCATGATTGGTGATCATTTTTCAAAA<br>GTCCTGTTCTTGCCTCCTAAATTTGCCCAAACACTGTCAGACTTGATTGATCAAAAAACT<br>CAGCTGGAGGATGCAAGTGGGCAGAGGTGGACGGTAACATTATCCAAATATGATGGGTCC<br>TTGGCTTTCCAACAGGGATGGCCTGCTTTTTCACTCGAACATGGTCTAGAAATTGGAGAT<br>TTTGTGTTATTCCATTACATAGAGCAATCTCACTTTGTTGTTCAAATCTATGGAAAAAGT<br>GGGTGTGAAAAACTCTATTTTTCGAAGGAAAATAGCAATCAGAAGAAACGAACAAGGGCT<br>AATAGAGATTCCAGTGCCAAAGATGAACAATGCCACACAATTAATGTTTCTATAAATTCT<br>ATGAACAAAGAGGGTTCAAATACTTCTGCTGTATCTGGTTCTGAAAGTGAAATAAGCCGA<br>AGGCTGCCAATGGCAACCACGTATACCACTTCAAACATTGATAACATCAATGAAAGGCTT<br>CATAATGTTCCCAGAACAAAATGCATCGAAGAACCATTTTATATGATTGATAGAGATTCT<br>GGGGTAGGACAAGTGGACCGCTTGAGCTCTTTATATGACTTGGCAGACTTTGAAATGCAA<br>AGGACACCCAGTGTTGATGGACCTAAAGAAGTTCTGGTAGAAGATGTAAGGCCCCTTTCC<br>CATGTTAATACAATGCCGCAATCTCAAGCTGAAGCCGAAATAGTGGATAAGTTACCTGTG<br>ACTGAAGAGGTAGTGAGCCAGACAACGCTTTCAGATGTACCTAACTTGCCAATTCTTGAG<br>AAAAATGAGTTTCAAATGATGGATAACGTGTCTAATCAAGATTATGGCAATGGCAACACT<br>TCTGGGCGAGAGCGGAGGAGCAAGACACGATATAGCAGCAATAAGGCTTTGATATCCCAG<br>AAAGATACTTCCCTCATGGAAAGGTGTCAAAGTATGCAACAGAAGTTTGCCGATGTGTCA<br>AAGTTCTGTGAATTTCCAGCCTCTGTTCAAACTTGGAATTGTCAATCTGGAGAGAGGATG<br>AAAGTGGTTAAAAAAGAGCAAGTGGAGATGAGGGCAAGGAAGATGATGCATGGAAAGTAT<br>CCAGAGATCAAGGAGTGTGACAGGAGTTCCAGTCCTTCCATGAAAGATAATGATCATGTT<br>TACAAAGTGGTGAAAACTGAACATGCAACGTCTACACTTTCAGTCAAAGTCCCTTTCTTG<br>GCGGTGCCGGGCAGGCTGTCTTTTCTTGAGTTGCCGAGTTGCTTGCCATCGGTTTCATTC<br>AGGGGAAGGCCCATAATGGAAAGGAAGGTATTAGTTCGCCTTCGAGACCCAGTATCTAGA<br>TTGTGGCCAGTCATCTACCATGAGAAACAGGGTATCAAAGTTTTGGGAAGTGGTTGGGAA<br>GCATTTTGCAAGGCAAATCGCATTCAGGTTGGAGATGAATGTGCTTTTGAAATTGAGGAT<br>GTGTTTGAGTGCATTTATAAAGTTAGTGTTGATAGGAAGTAA	1542bp	MGWPVATSFFKIMIGDHFSKVLFLPPKFAQTLS<br>DLIDQKTQLEDASGQRWTVTLSKYDGS<br>LAFQQGWPAFSLEHGLEIGDFVLFHYIEQSHFV<br>VQIYGKSGCEKLYFSKENSNQKKRTRA<br>NRDSSAKDEQCHTINVSINSMNKEGSNTSAVS<br>GSESEISRRLPMATTYTTSNIDNINERL<br>HNVPRTKCIEEPFYMIDRDSGVGQVDRLSSLY<br>DLADFEMQRTPSVDGPKEVLVEDVRPLS<br>HVNTMPQSQAEAEIVDKLPVTEEVVSQTTLSD<br>VPNLPILEKNEFQMMDNVSNQDYGNGNT<br>SGRERRSKTRYSSNKALISQKDTSLMERCQSM<br>QQKFADVSKFCEFPASVQTWNCQSGERM<br>KVVKKEQVEMRARKMMHGKYPEIKECDRSSS<br>PSMKDNDHVYKVVKTEHATSTLSVKVPFL<br>AVPGRLSFLELPSCLPSVSFRGRPIMERKVLVR<br>LRDPVSRLWPVIYHEKQGIKVLGSGWE<br>AFCKANRIQVGDECAFEIEDVFECIYKVSVDRK	513	The theoretical size of CsREM1 protein is 58.36 KDa. The theoretical size of the CsREM1-GST recombinant protein is 85.33 KDa.<br>The actual size of the CsREM1-GST recombinant protein is between the 110 KDa and 75 KDa protein markers, which is consistent with the theoretical size (Figure S5).
CsWRKY50	CSS0032848	ATGGATTCTCCGGAATCGGATAGTGACTATGCTGCAGCAGCAGCAGCAGCCAACCAGTCG<br>AATTTCGAGCTTTCTGATTTCTTCGAGTTTGATGAGGAGTGGGCTGAGGGAGATCATCCA<br>CCACCACCAACACCGATGGTGGTTTCCGGGTATCCCCAAATGAATCCAGTTTATGCTGCA<br>GGATCAGCAGCGAGTCACGGAATCAATGATGCAGCCAGGGGAAGCAGTAGCCACCTCAGT<br>ATCTATGAAGGAACTAGTATCATAGGGGACAGTGGTAGGAGTTGTGGGAGGGAGAAGAAG<br>GAAGTGAAAGAGAAAGTTGCTTTTAAAACACAATCAGAGATTGAAATACTTGATGACGGC<br>TTCAAGTGGAGGAAGTACGGCAAGAAGATGGTCAAAAACAGCCCAAATCCAAGGAACTAT<br>TATAGGTGTTCAGCAGAAGGATGCCCAGTGAAGAAGAGAGTTGAAAGAGACAGAGATGAT<br>CCGAGGTACGTCATAACTACTTACGAGGGTGTCCATAACCACCAAGGTCCTTCTTCTACT<br>ACTATTACTACCAAGTTCTGA	561bp	MDSPESDSDYAAAAAAANQSNFELSDFFEFDE<br>EWAEGDHPPPPTPMVVSGYPQMNPVYAA<br>GSAASHGINDAARGSSSHLSIYEGTSIIGDSGRS<br>CGREKKEVKEKVAFKTQSEIEILDDG<br>FKWRKYGKKMVKNSPNPRNYYRCSAEGCPV<br>KKRVERDRDDPRYVITTYEGVHNHQGPSST<br>TITTKF	186	The theoretical size of CsWRKY50 protein is 21 KDa. The theoretical size of the CsWRKY50-GST recombinant protein is 48 KDa. The actual size of the CsWRKY50-GST recombinant protein is between the 63 KDa and 48 KDa protein markers, which is slightly larger than the theoretical size. However, the CsWRKY50-GST protein was detected by Western Blot experiment using GST-Tag antibody. It is speculated that it may be due to the formation of protein dimers or post-translational modification, resulting in a larger size than the theoretical size (Figure S5).

Note:Those date were from our own lab.

Supplementary Table S5. The promoter sequences of *CsTSI* and *CsREM1* .

Name	Promoter region sequence	The number of nucleotides in thePromoter region sequence
ProCsTSI	AGACACACCTAACATAAATACACCTAAAAAGTAAAATAATTGTAAAAAAAATCTTTTAAAAATTCACTAAATATCAAAAATATAAAAAAAATACAAGAATTCAGTA<br>CATTTTTTGTATTTTTCTGTATTTATTAATTTTTAAAAATATTTTTTCACAAATTTTTTACTTTTTAGATACATCTAAGTAAGGTGCATCTAAAAAAGTAAAAAAATGT<br>TTAAAATTTAAAAAATTCACTAAAAACAAAAAAATACAAAAACTAAAAAAATTAATTTTCAAAAAACTTAATTCAGAAAACTCAAACCAAACACAGTGACTAGTTT<br>TTAGGTATTTTGTCCTTTACTTTATTAAAAAATAATAAAAATATAAAATACGTAATTAAATTGAGAAATGTTATGCAATACACACGGTATAAAATTTATATAAAATT<br>ACGTAAATTTTCACAAAATAATTATACAAAAATACGTAAATTAAACATAAATTTATGTAAATTAATGAAATCTATGTGATTATGCGAATTTGTGTTGAATTTACACA<br>TTTTTATAAGATCAATTATGTAAATTCATAAAAACTTATGTAATTTTATGTAGAGTTATACATGGTGTGCAATGCACACTGAAATTTTTTCCAATTAAATTTATTAGA<br>AAATATTTAGAGTGTACATAATAATATAATTGGACATACTTTTAGTGAAAATAATGAAAATAACCCATAAAAACATCTTTATTGAGTTCATGGGGTATGTTTGGTTC<br>AATTTCTTGTAAATTAAAAATTAATAATAAAAAGTAAAAATTAAAAGCATCACTCTCTTAATTTTTAAATAATTATTATTTTTTCAGTTTATTAAAATTATTTATACTA<br>GATAAGCTATATACATAAGTTACATTAATTTGTTTAAACAGTGTCACACATACTTTCAAAATAAAAAATATATATAAACTGCACTAAACAACCCTATTGTGTTGACT<br>AATATATTTTTGAAAATCTAATAGGTAAGGTCTTGTTTGGGGATATAATTAAAAAGTTTTTATAAATTGTCACAATATTATTTTATTTATATTTTTATAAAAAAATTAAG<br>AAAGTAACTAAAATAATTAATTTCCAAACGAGTTATTAATTTTGACTTATTATGAAAATAAGAAAAAAAAAATCAACTTTTTAATTTTTTTATAATAAAAATAAAAAT<br>ATAATATGATCCAAATAAAATAATAAACTTATTTTTTTTTAGTTGTCAAAATGTGGGTACACAGTAGGGATGTAAGAAAAATCGAAAAAATCGACCAAATCGATTG<br>AACCAGACCAAACCGGCTATATTTGATCTGTTTTTTAACAATAATCGGTCTGTGCGGTTTTCAAATTTGAATTTTTTGGATTTTCGGTTCGGTAACGGTTTGAGCACC<br>TTGTGCACCGATCTAAACCAAAACCGACTGCTTCAACATATATTGTATTTATAATATATATATATATATATAATCTATTAATCTAATGATATTAATAAGTATAATAAA<br>TTATTAACCTTAATTAATTAACCTTATCATCTTCATAACCTAAGTTTGTAAGCTCTTATTATATATAATTTTAACTCTAACTCATTATTCTTGAAACTTCAGTATTTTTA<br>AACTTTTTTTTGTTTTTATTTTTATTAATTTAGGAATTAAAAAGTATGTATTAAATAGATGAATTGGATACGTTTGAAATGGTAAACAAATAACTAATTTAATATTTTT<br>ATATAGATTAATTATTATATTTAAAAATGAGTTTGAAAAATTTTAAACAGATTAAATCGCACCAAACAGAACTGTTTCAATTAGTTTAATTCAGTGTAATTTGATGTG<br>AGAACTGTATAAATGTTCAAAATAATAAAATTGACATTAATAATTCAATGTAATTTTTTATTAAAATACCGACGGTATCAAACCGATTACAACATGCTACACAGACT<br>CGTAAAAACAGAAGTCATTAATTCGAGGGGCTGCTCTGCTCTGGACTCTATAAAACCCACTC	2000bp
ProCsREM1	AAACAAGGCCTTAGAATGGGCCATGTTAGTATGTTGGAAACATGCTTTTTAGTAATTTTTTGGTTTTTTGTCTATAATATGACATAAAAAAAAAACTAGTATGTTCGG<br>GATGACTTTTTAATTTTTTATGAAAAGATGTGCAAAAAGTAAAAAGATAAGGGAATCTTTAACTTTTTTGTATAAGTTGTTGGCTTTTTTTTCTTTTCTTTTTTTAAAG<br>TAACTTTTTAACTTTTAAGCTCAAAAGTGATGTTGCGAAACTAGGCAGTATACACAAAATCACAAAAGCCACAACTACATCCTTTTAGTCGTAACTAAAAAAGCAT<br>AATGGGTCATAATCAAACAGAAAGACTTTTTTTTTTTTAACGTAATCACAATAACAATCCCAATAGAAATAATCAACATCACAATCAGTCATAACACAAAAAATGA<br>ATGTATTTTATAAAATTTCTCTTTAGAATGACATACTATAAGATAATAATTATATATGAAGAAAAGGAGAAAAAGGAGGAGAGAGCATAGCAATTGACCTTGGTGA<br>CAGAGAGTAATGCGAGCGAGGAATTGAGAAGTGCTTTGTTTTTGGTACAGAACTTTAACAATGGAGGGTTTGGGGTTTGAATTAAACCCTAGTTTTTTTCATACTCT<br>CTCTCGATATCCCTCTGTTCTTTCCAAAGTTCTACTAGTCTCTAGTGTAAAGTGTAGTTCCCTCCTTTCAACGAAACGCCTCTCTCCCTAGCATAATATACATTCATT<br>CTAAACTAGGGACATGCTGGGACACGCTGAAGATACGTTAAATTTTTTACTAAAATAGCATACCTAAAGTTGATAAATTTAATAAAATTAAATATTTGACATAAATA<br>AATAAATATTTAAAAACATGACATATAATAAATATACAAAATTATATGTTTTAAACATGTCCCCAACGTGTGTTGTCTTGCATTTTTTAAAAATTTGCGTGTCTGTGT<br>CCGTGTCGGTGCTTCTTAGAAGAAGCAAACTCAATTGGTTGCTCCAAAATATGTGCCCATTGTTGAGAAGGAATCGGCATGCCAAAATCTAACTAATGGTCTCTTTC<br>ACCCTAATTGATATTAATTTCCTCTGATACAAAGAAGCACGAGCACTTCTAAAAATGTGGTGTGTAGATGTCGTGTCGTGTCAGACACCGACACTTGTCAAAAAGTG<br>TCTTATTTTAAGGAAAAAAATAGATCCGGACACTTGGAGGACACGTGTCCATTGATTAAACGTATGAAACTAATTATAAGGTTAAACTAAATGACATAGGATACGG<br>TAATAGAAGAAAAACAATATAAATTTATAAAATTAAGTGTTATATTGTATTTTTTTTAATTTTCTTTAAATGATATATTCATATATATGTCATATAATTAATATATAAT<br>TAATATATTTTGTACGTGTTCTCCATGTGCCGTGTCATGCATTTTTTGAAAATCGATATTGTATCTGTGTCTATGCTTCTTAATATATATATATATATATAGATCTACG<br>TGGTTACATTTTGTGTGTTTGTAAACCAAGGTGTTAGAAGTAGTGCGAAGGAGGGGGCATGTTTGTGGGGAAGAATGGATGATGGGTAAAAATGACTTTTTGTCTAT<br>GTTTTTGACTTACTAATAATTAAATTCCTATACATAGATGTTCGAAGAAATATTTTCATTAAACGCACTTATCTATAATAAATTTGTTATTTATTGTTGTAAATGTAAT<br>AATCTCTATATTTATTTATGATCAATCTTTGTTTGAGAACCCTGGTTGTTTTTTATTTGAATTATATTATTTTATGTTTTTTATGAAATCACTTCATGAGTAAATTTGAA<br>AGCTCTTACGGAGACTTTGAATGCCTTATTATATTCGTGGCTAAATGGTTTGCCTTCTTTGGACCTTTTGCCCTCCCCTTTTTTTTTTTTTGTAAAACTTCTAATTGATT<br>TTGTTTTGTTTCTTTGTATATATAAGTTTACAGCAGAGGGAATTTCATCAGTGGAGAAGAG	2000bp
